# Roles of CD34^+^ cells and ALK5 signaling in the reconstruction of seminiferous tubule-like structures in 3-D re-aggregate culture of dissociated cells from neonatal mouse testes

**DOI:** 10.1371/journal.pone.0188705

**Published:** 2017-11-30

**Authors:** Shin-ichi Abe, Kazuko Abe, Jidong Zhang, Tomoaki Harada, Go Mizumoto, Hiroki Oshikawa, Haruhiko Akiyama, Kenji Shimamura

**Affiliations:** 1 Center for General Education, Kumamoto Health Science University, Kumamoto, Japan; 2 School of Basic Medical Sciences, ZunYi Medical University, Zunyi, Guizhou Province, China; 3 Department of Biological Science, Graduate School of Science and Technology, Kumamoto University, Kumamoto, Japan; 4 Department of Orthopaedics, Graduate School of Medicine, Gifu University, Gifu, Japan; 5 Institute of Molecular Embryology and Genetics, Kumamoto University, Kumamoto, Japan; University Hospital of Münster, GERMANY

## Abstract

Tissue reconstruction *in vitro* can provide, if successful, a refined and simple system to analyze the underlying mechanisms that drive the morphogenesis and maintain the ordered structure. We have recently succeeded in reconstruction of seminiferous cord-like and tubule-like structures using 3-D re-aggregate culture of dissociated testicular cells. In testis formation, endothelial cells that migrated from mesonephroi to embryonic gonads have been shown to be critical for development of testis cords, but how endothelial cells contribute to testis cord formation remains unknown. To decipher the roles of endothelial and peritubular cells in the reconstruction of cord-like and tubule-like structures, we investigated the behavior of CD34^+^ endothelial and p75^+^ cells, and peritubular myoid cells (PTMCs) in 3-D re-aggregate cultures of testicular cells. The results showed that these 3 types of cells had the capacity of re-aggregation on their own and with each other, and of segregation into 3 layers in a re-aggregate, which were very similar to interstitial and peritubular tissues *in vivo*. Observation of behaviors of fluorescent Sertoli cells and other non-fluorescent types of cells using testes from *Sox9-EGFP* transgenic mice showed dynamic cell movement and segregation in re-aggregate cultures. Cultures of testicular cells deprived of interstitial and peritubular cells resulted in dysmorphic structures, but re-addition of them restored tubule-like structures. Purified CD34^+^ cells in culture differentiated into p75^+^ cells and PTMCs. These results indicate that CD34^+^ cells differentiate into p75^+^ cells, which then differentiate into PTMCs. TGFβ signaling inhibitors, SB431542 and ALK5i, disturbed the reconstruction of cord-like and tubule-like structures, and the latter compromised re-construction of interstitial-like and peritubular-like structures, as well as the proliferation of CD34^+^, p75^+^, PTMCs, and Sertoli cells, and their movement and differentiation. These results indicate that CD34^+^ cells and signaling through ALK5 play pivotal roles in the morphogenesis of interstitial-like, peritubular-like and cord-like structures.

## Introduction

Testicular morphogenesis is initiated from bipotential gonads at 10.5 dpc (days post coitum) by the expression of Sry, a testis-determining gene in pre-Sertoli cells (SCs), followed by sequential steps toward formation of seminiferous cords and tubules [1–3]. The process toward cord formation starts from the aggregation of SCs around germ cells. During prenatal testes p75 neurotropin receptor (p75NTR) ^+^ cells occupy the whole intertubular compartment around testis cords, and progressively concentrate around a PTMCs layer that surrounds each testis cord [4]. The high expression of p75 as a layer around each cord decreases and disappears at puberty [4]. Concomitant with the formation of the cord, interstitium comprising Leydig cells (LCs), vascular cells and mesenchymal cells is formed outside the cord. Critical roles of mesonephroi in the formation of the cord have been reported by many investigators [1–3]. When XY gonads are cultured without mesonephroi or separated from it by a permeable filter, they do not differentiate into cords [5], indicating that migration of some cells from the mesonephroi into gonads is indispensable for the formation of cords. The migrating population has been reported to include endothelial, vascular-associated cells and PTMCs [5–8]. Some studies suggested that PTMCs are derived from the migrating population and contribute to the formation of the cords. Important roles of co-operative interactions between SCs and PTMCs in the formation of the cords in *in vitro* cultures were also reported [9–11]. ECM components are cooperatively produced and deposited by SCs and PTMCs, e.g., laminin by the former and fibronectin by the latter [11, 12], and the ECM promotes the testicular cord formation [10, 13]. On the other hand, Cool et al. [14] showed that PTMCs labeled with EYFP do not migrate from mesonephroi to the XY gonad, and suggested that endothelial cells migrate and are required for epithelialization of testis cords. Combes et al. [15] also concluded that immigrant cells are endothelial cells, not PTMCs or other interstitial cells, and that the migration of endothelial cells is required for testis cord formation, since blocking antibody to VE-cadherin impedes endothelial cell migration and disrupted the development of testis cords. However, how endothelial cell migration contributes to the formation of the cords remains to be clarified.

Various factors have been reported to be involved in cord formation [1–3]. For example, TGFβ signaling is required for testis development *in vivo* [16–18] and *in vitro* [19]. Addition of SB431542 (an inhibitor of ALK4, ALK5, and ALK7) or ALK5i (an inhibitor of ALK5) to organ cultures of E11.5 XY gonads disrupts testis cord formation [19]. The TGFβ superfamily comprises more than 40 ligands, including TGFβ1–3, activin A and B, inhibin A and B, and bone morphogenetic proteins (BMPs) [16]. These ligands form dimers and bind to a type II receptor which has constitutive serine/threonine kinase activity, and which recruits and phosphorylates a type I receptor with Ser/Thr kinase activity, resulting in the activation of downstream Smad-dependent signaling cascades [20]. There are many type I receptors, such as ALK1-ALK7, and many type II receptors, such as TGFβRII, ActRIIA, ActRIIB, and BMPRII. Each ligand recruits a distinct set of type I and type II receptors. TGFβ1, TGFβ2 and TGFβ3 proteins are expressed by SCs, some interstitial cells (including LCs), and gonocytes in a stage-dependent manner [21–23]. The functions of TGFβs have been examined by gene knockout for each TGFβ isoform, resulting in the finding that the null-mutants for each TGFβ isoform show no major morphological defects in gonad development in mid-embryonic stages, but die in late embryonic or early postnatal stages [17]. Double-knockout mutants for various combinations of the TGFβ isoforms die early in embryonic development, making it difficult to analyze the effect on gonadal development [24]. Studies of TGFβr3-null mutant embryonic testis have indicated that this isoform functions in normal seminiferous cord formation and fetal LC development [18].

Tissue reconstruction *in vitro* can provide, if successful, a refined and simple system to analyze the underlying mechanisms, including key ligand(s), their receptors, cellular adhesion and motility, and signaling pathways that drive the morphogenesis and maintain the ordered structure. Reconstruction of testes has been hampered by difficulty in developing an appropriate culture system: Seminiferous cord-like structures in which polar SCs with columnar shape are united by tight junctions are formed in culture [9–11], but further morphogenetic events leading toward the formation of seminiferous tubule-like structures were not achieved in the past, unless the *in vitro*-formed cord-like structures were transplanted under the backskin of immunodeficient nude mice [25, 26]. Recently Alves-Lopes et al. [27] developed a novel three-dimensional multilayer model, the Three-Layer Gradient System, in which rat testicular cells placed in Matrigel were sandwiched by two Matrigel zones. The cells formed spherical testicular organoids with a functional blood-testis barrier and germ cell establishment and proliferation. Pendergraft et al. [28] also reported the development of a three-dimensional in vitro human testis organoid culture system in which multicellular human testicular organoids composed of spermatogonial stem cells, immortalized Sertoli, Leydig and peritubular cells were created in hanging drop culture plates. The results showed continuous production of testosterone, upregulation of postmeiotic genes, and response to chemotherapeutic drugs in a dose-dependent manner, indicating a potential to be used as a novel testicular toxicity-screening tool and in vitro model for human spermatogenesis. Seminiferous cord-like or tubule-like structures, however, did not seem to be re-constructed. We have recently succeeded in the reconstruction of seminiferous tubule-like structures *in vitro* using a 3-D culture of dissociated testicular cells from neonatal mouse [29]. When testicular cells dissociated from neonatal mice are cultured in the presence of KnockOut Serum Replacement (KSR) within a collagen matrix, seminiferous tubule-like structures are formed, accompanied by the attachment of PTMCs around the SC re-aggregates, the differentiation of SCs (as indicated by expression of the androgen receptor), the formation of a blood-testis-barrier between the SCs, SC elongation in the radial direction and lumen formation.

In the current study, to decipher the roles of endothelial cells and peritubular cells in the reconstruction of seminiferous cord-like and tubule-like structures, first we investigated the behavior of CD34^+^ endothelial cells as well as p75^+^ cells and PTMCs in 3-D re-aggregate cultures. The results indicated strong interactions among these 3 types of cells and their pivotal roles in the formation of seminiferous cords and tubules. Secondly, behaviors of fluorescent SCs and other non-fluorescent types of cells were observed using testes from *Sox9-EGFP* transgenic mice [30]. The results showed dynamic behavior of SCs and other types of cells and their segregation. Thirdly, to test whether TGFβ signaling is involved in the reconstruction of seminiferous cord-like and tubule-like structures from dissociated testicular cells, we examined the effects of SB431542 and ALK5i on the reconstruction. Our results indicated that signaling through ALK5 is indispensable for the morphogenesis of seminiferous cord-like structures.

## Materials and methods

### Mice

C57BL/6J mice were purchased from CLEA Japan, Inc. (Tokyo, Japan). C57BL/6J and *Sox9-EGFP* transgenic mice [30] were maintained on a 12-h day/12-h night schedule at constant temperature and humidity in the Center for Animal Resources and Development of Kumamoto University, and at Kumamoto Health Science University according to protocols for the animal experiments approved by the respective Institutional Animal Care and Use Committees. Total number of mice used in this study was more than 60.

### Re-aggregate culture of dissociated testicular cells

Four to 8 mice of 10- - 12-dpp were used per experiment, depending on the number of male pups which had been born from one or two mother mice. Dissociation of the testes, re-aggregation of the dissociated cells, and culture in a 3-D system inside a collagen matrix were performed as previously reported [29] with some modifications. Briefly, after the tunica albuginea was removed, whole testes from 4 mice were put in 3 ml of RPMI medium, cut into several pieces using scissors, and enzymatically digested with collagenase (type IV, Worthington, final concentration 0.08%) and hyaluronidase (Sigma, final concentration 0.1%) for 1 hr at 25°C. If 6–8 mice were used, testes were distributed to two dishes with 4 ml RPMI medium plus enzymes. The testicular cell suspension was treated with DNase I (Sigma, final concentration 0.01%) for the final 5 min, followed by thorough dispersal by pipetting and filtering through nylon gauze (50 μm). The dissociated cells were washed in medium and then re-aggregated by rotation culture (60 rotations/min, R-30, TAITEC, Koshigaya, Japan) for 1 hr at 25°C. The cell suspension was divided into 10–20 siliconized tubes (0.5 ml) (Assist, Japan) so that one tube contained 7–8 x 10^5^ cells, followed by centrifugation at 230 *g* for 5 min (Sigma-Kubota, #3615, or Eppendorf Centrifuge 5418R). Each cell pellet was sucked up by a pipetman with 70 μl collagen (Cellmatrix Type I-P, Nitta Gelatin Co., Japan, final concentration 2.4 mg/ml) in RPMI-1640 medium (Wako, Japan), pH 7.4, and put on a Nuclepore filter (Whatman, USA) in a 35-mm plastic dish (Falcon 351008). The pointed end (ca 1mm) of yellow tips had been cut off. After the collagen hardened (20 min), the filter was floated up by pouring medium (2 ml, RPMI-1640) with or without supplementation with 10% KSR (Gibco, USA). One pellet embedded within collagen was put on a filter in one dish. The dishes were kept at 34°C in a humidified incubator (APM-30D, ASTEC, Japan) with an atmosphere of 50% O_2_ and 5% CO_2_. Stock solutions of SB431542 (CAY) and ALK5i (Wako) were made by dissolving them at 10 mM in DMSO and 15 mM in methanol, respectively. SB431542 and ALK5i were added to the medium at final concentration 20 μM and 15 μM, respectively. As controls, 0.2% DMSO and 0.1% methanol was found to have no effect on the culture of testicular re-aggregates. At least three experiments were performed for each condition.

### Re-aggregate culture of dissociated testicular cells deprived of interstitial and peritubular cells

To fractionate testes into two fractions, one rich in seminiferous tubules and another one rich in interstitial and peritubular cells, testes cut into 3–4 pieces were treated with 0.08% collagenase, 0.013% hyaluronidase and 0.01% DNase I for 30 min at 25°C, and gently pipetted up and down about 50 times using a pipetman with a blue tip (whose pointed end (ca 1mm) had been cut off and which had been coated with 0.5% BSA by sucking up and down several times using a pipetman), resulting in dispersal of the testes into seminiferous tubules. After a further 30-min treatment with the same enzyme solution as above, the solution containing seminiferous tubules was further pipetted gently up and down about 50 times using a pipetman with a shortened, widened, BSA-coated blue tip made as described above. The solution was filtered through 50-∝m nylon mesh once, and the residue on the filter was the tubule fraction, while the filtrate was the interstitial and peritubular fractions. The residue was washed with RPMI medium several times, and then the filter was reversed and dipped into RPMI medium. The tubules thereby recovered by dipping in the medium were enzymatically digested with collagenase and hyaluronidase for 1 hr at 25°C, as in the above section, followed by thorough dispersal by pipetting and filtering through 50-μm nylon mesh. The dissociated cells were washed, re-aggregated by rotation culture, and the cell pellets in Eppendorf tubes were embedded within collagen matrix as described in the above section (Re-aggregate culture of dissociated testicular cells). On the other hand, the filtrate was further filtered through a 30-μm nylon mesh, and the cells in the filtrate were collected by centrifugation and washed. These cells were a fraction rich in interstitial and peritubular cells.

### Purification of CD34^+^ cells and culture on coverglasses

The fraction rich in interstitial and peritubular cells isolated as described in the above section was washed in RPMI-1640 medium, collected by centrifugation at 230 *g* for 5 min, and dispersed in 100 μl of RPMI-1640 with L-glutamine, Hepes and Na pyruvate containing 0.5% BSA. Anti-mouse CD34 conjugated with biotin (2 μl, eBioscience, RAM34) was added to the cell suspension and it was incubated in a refrigerator for 10 min, and washed twice with 2 ml of BSA-containing medium. Then anti-biotin microbeads (20 μl, Miltenyi) were added to 80 μl of cell suspension in BSA-containing medium for 20 min, and washed twice. The cell suspension in 500 μl of BSA-containing medium was applied to a MACS column (MS column, Miltenyi). The CD34^+^ fraction was purified again by application to a new column. One hundred microlitters containing 3–5 x 10^5^ cells were put on each coverglass (13 mmø, Matsunami, Japan), which had been treated with 0.1% poly L-lysine and then coated with 0.06% collagen (type I-C, Nitta Gelatin Co., Japan). The coverglasses placed in a 35-mm diameter plastic Petri dish (Falcon #351008), were incubated at 34°C in a humidified incubator. Most of the cells settled on the coverglasses within a few hours and extended their cytoplasm to a small extent. The cells on three coverglasses were fixed with 4% paraformaldehyde (PFA) (Wako, Japan), and the rest were divided into half to which RPMI-1640 + 10% KSR was added, and the other half to which StemPro medium {StemPro-34 (Gibco) 20 ml + RPMI-1640 20 ml + fetal calf serum (high performance, Gibco, 10 ml)} [31] was added, and cultured for 3 days in the absence or presence of ALK5i.

### Immunofluorescence

Cultured re-aggregates were fixed in 4% PFA and embedded in paraffin. Sections (5 ∝m) were boiled in 0.01 M citrate (pH 6.0) with 0.1% Tween 20 for 10 min, washed three times in 0.1% Tween-20/PBS, transferred to blocking solution containing 5% BSA and 5% horse serum (Sigma) or goat serum (Invitrogen) in 0.1% Triton X-100/PBS for 1 hr, and incubated with primary antibody at 4°C overnight. After washing, the secondary antibody was added, and the sections were incubated for 2 hrs at room temperature. The antibodies used in this study are listed in Table 1. Microscopic images were obtained using a CCD camera (DP72, Olympus, Tokyo) mounted on a fluorescence microscope (BX60, or BX61VS-ASW, Olympus).

**Table 1:** The antibodies used for immunofluorescence.

Antibody		Vendor		Catalog no.	Working dilution
goat anti-GATA4		Santa Cruz Biotechnology		Sc-1237	1:200
mouse anti-α-SMA (mAb)		Sigma-Aldrich		A 2547	1:10000
rabbit anti-laminin		Sigma-Aldrich		L9393	1:200
rat anti-mouse CD34		BioLegend		119301	1:200
rabbit anti-CD34 (mAb)		Abcam		ab81289	1:200
rabbit anti-p75 NGF Receptor (mAb)		Abcam		ab52987	1:100
mouse anti-PCNA (mAb)		Santa Cruz		SC-56	1:200
mouse anti-PCNA (mAb)		Abcam		ab29	1:200
rabbit anti-PCNA (mAb)		Abcam		ab92552	1:200
donkey anti-rabbit Alexa 594		Abcam		ab150076	1:200
donkey anti-rabbit Alexa 488		Abcam		ab150073	1:200
donkey anti-mouse Alexa 594		Abcam		ab150108	1:200
donkey anti-mouse Alexa 488		Abcam		ab150105	1:200
donkey anti-goat Alexa 594		Abcam		ab150132	1:200
donkey anti-rat Alexa 488		Invitrogen		A21208	1:200

Cultured cells on coverglasses were fixed in 4% PFA. Antigen retrieval was done by incubation with 100% methanol (-20°C) 10 min, and 0.3% Triton X-100 for 10 min.

### Terminal deoxynucleotidyl transferase-mediated dUTP-biotin nick end-labeling (TUNEL) staining

The sections from paraffin-embedded testes were subjected to TUNEL staining using the Apoptosis In situ Detection Kit (WAKO, Japan) according to the manufacturer’s instructions. The number of positive cells was counted among 1700–4000 cells in 2–4 sections, each derived from every five sections, in the presence or absence of ALK5i in one experiment. The number of dead cells was expressed as the percent of cells (means ± S.E.M.)/experiment from three independent experiments.

### Proliferative activity of SCs, CD34^+^ cells, p75^+^ cells and PTMCs

To assess the proliferation of SCs, CD34^+^ cells, p75^+^ cells and PTMCs, the sections were stained with a mouse anti-PCNA antibody, and with an antibody against GATA4, CD34, p75 or α-SMA, respectively, and their images were obtained using a fluorescence microscope.

The proliferative activities of SCs, CD34^+^ cells, p75^+^ cells and PTMCs were expressed as the percentage of cells that were double-positive for PCNA and GATA4, CD34, p75 or α-SMA, relative to the total GATA4- or CD34-, p75- and α-SMA-positive cells, respectively, which were counted in 3 photographed areas selected at random in a section x 3 sections. These 3 sections consisted of every eighth section (40 μm apart) in a sample. The average values (means ± S.E.M) from 3 independent experiments were calculated.

### Differentiation capacity of purified CD34^+^ cells

The differentiation capacity of purified CD34^+^ cells cultured for 3 days in the presence or absence of ALK5i was evaluated by performing immunofluorescence analysis assessing whether CD34^+^ cells had changed to cells expressing p75 and/or α-SMA. When the expressions of CD34 and p75 in combination were examined, all the cells in a marked area were classified into cells which expressed only CD34, those which expressed both, those which were positive only for p75, and those negative for both. The percentage of each group was obtained when the total number of cells was assumed as 1.0. Similarly when examining the expressions of p75 or α-SMA, or their combination, cells were classified as cells expressing only p75, or only α-SMA, those double-positive for p75 and α-SMA, and those negative for both antibodies. For α-SMA and CD34, the percentages of cells positive for their expression were obtained in the same way. The average values (means ± S.E.M) from 3 independent experiments were calculated.

### Time-lapse imaging of re-aggregate culture

Ten-12-dpp *Sox9-EGFP* testes, which were identified as positive for EGFP expression using an epifluorescence stereomicroscope (Olympus SZX12), were dissociated and re-aggregated as described in “Re-aggregate culture of dissociated testicular cells”. The cell pellet formed by centrifugation was cut into smaller pieces using needles, sucked up using a pipetman with a shortened, widened, yellow tip which contained about 200 μl of medium, and placed in several areas on a cell culture insert (Falcon, REF353090) whose cylinder was cut at a height of about 5 mm so that it could be fitted in a glass-bottom dish {3910-035-MYP, 27 mm (No.1), IWAKI}. After removing the medium on the culture insert, collagen solution (ca 200 μl) was poured into several areas among and on the cell pellets. After letting it stand for 20 min, the culture insert was put into a glass-bottom dish. Medium (1.5 ml) was poured into the outer area of the dish. The culture dish was put in an incubator for 1 hr, and then placed on a time-lapse disk-scanning confocal microscope (Yokogawa Electrics, CV1000). Time-lapse imaging was performed over 3-7-days period with a non-immersion x10 objective lens at 34°C with 50% O_2_ and 5% CO_2_ in a humidified environmental chamber using a 488 nm laser. Confocal optical Z-sections were collected every 14 μm throughout 280 μm at 30-min intervals. All time-lapse images presented in this report are maximum-intensity projections (MIPs) except otherwise stated.

### Statistics

Cell proliferation activities and their comparison between culture days in the absence (C) and presence (K) of KSR were analyzed by *t-* test. Bonferroni correction was used for multiple comparisons. *P* values that were less than 0.05 were considered statistically significant.

## Results

### CD34^+^ cells, p75^+^ cells, and PTMCs re-construct interstitial-like and peritubular-like tissues during re-aggregate culture of dissociated testicular cells

**Fig 1:**
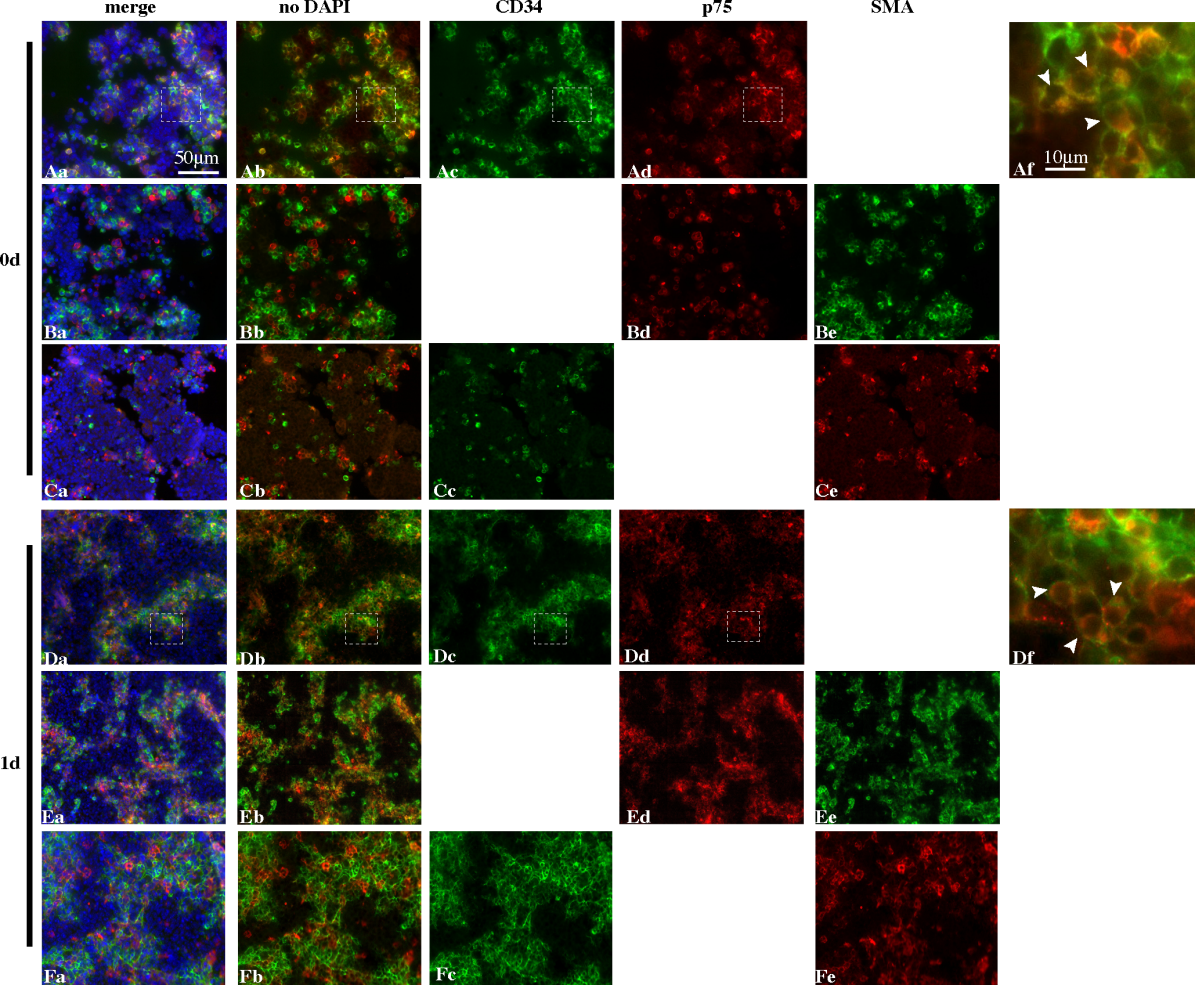
Re-construction of ordered structure of peritubular-like and interstitial-like tissues in re-aggregate culture of testicular cells (day 0 –day 1). Expression of α-SMA, p75 and CD34 are shown as a pair of the three in each row, and in three rows on day of 0 (Aa-Ce) and 1 (Da-Fe). Af and Df show magnified view of the area depicted in Aa-Ad and Da-Dd, respectively, which contained cells positive for both antigens {arrowheads in (Af) and (Df)}.

**Fig 2:**
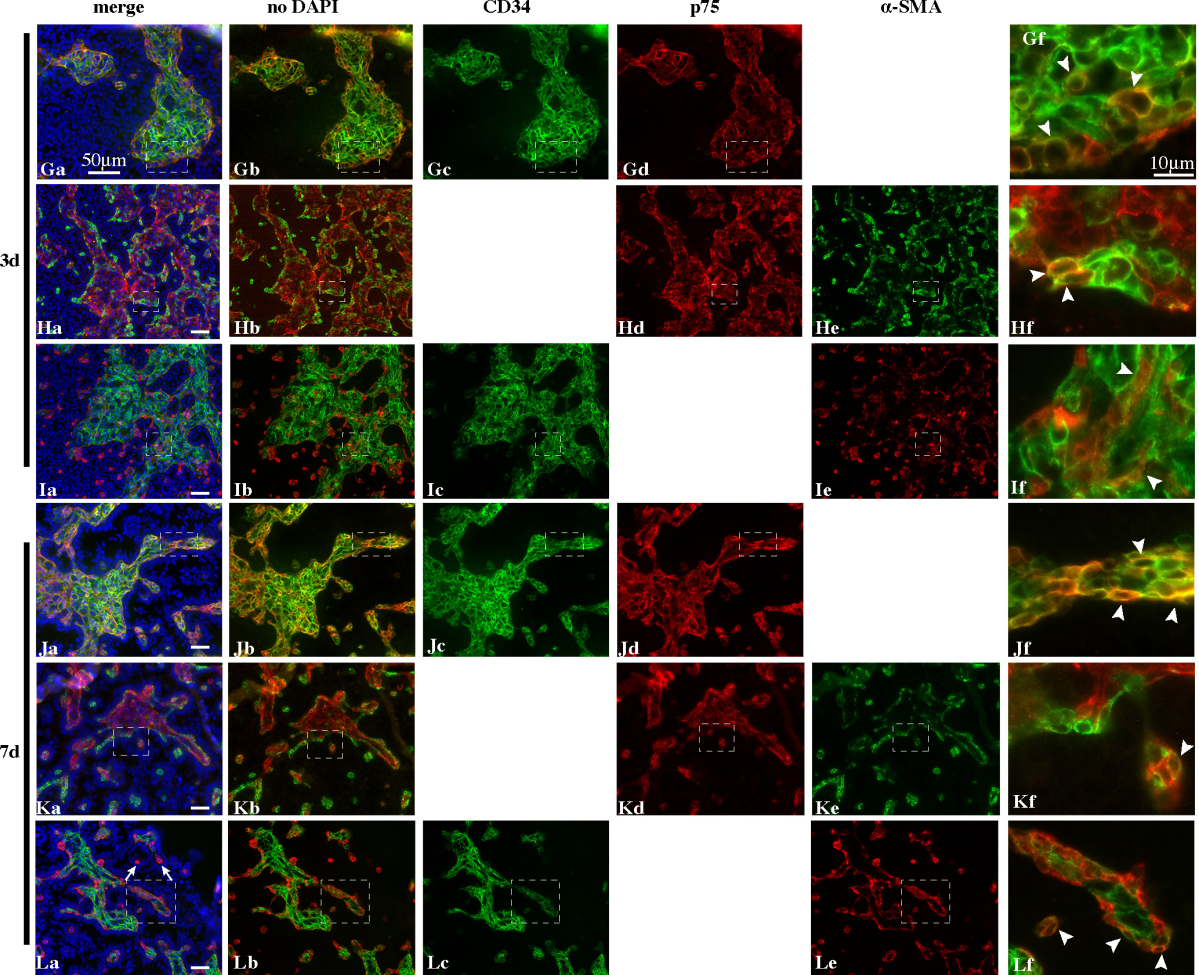
Re-construction of ordered structure of peritubular-like and interstitial-like tissues in re-aggregate culture of testicular cells (day 3 –day 7). Same as in Fig 1, but on day 3 (Ga-If) and day 7 (Ja-Lf). Arrows in (La) show one or a couple of PTMCs around which SCs were assembled like a rosette.

In the current study, in order to determine the roles of endothelial cells and peritubular cells in the reconstruction of seminiferous tubule-like structures we observed the behavior of CD34^+^ cells, p75^+^ cells, and PTMCs by immunofluorescent staining with antibodies specific for each cell type on day 0, 1, 3, 5 and 7 of culture (Figs 1 and 2). As we have previously reported [29], at the start of the re-aggregate cultures, SCs form many small re-aggregates (30–70 ∝m in diameter) throughout the testicular cell-re-aggregates, while round PTMCs are randomly distributed. On day 0, most of the CD34^+^ and p75^+^ cells existed as re-aggregates on their own, while many PTMCs were distributed as single cells or re-aggregates consisting of a small number of cells, and the three types of cells were observed to exist closely to each other (Fig 1Aa-Ad, 1Ba-Be and 1Ca-Ce). It is noteworthy that some cells expressed both CD34 and p75 simultaneously, though the expression level of each antigen varied depending on the cell (white arrowheads in Fig 1Af and S1Ag-Ai Fig). In contrast, few PTMCs seemed to show simultaneous expression of α-SMA with CD34 or p75 (Fig 1Ba-Be and 1Ca-Ce). On day 1, re-aggregates of PTMCs, p75^+^ cells and CD34^+^ cells each became larger and intermingled with each other (Fig 1Da-Fe). While most PTMCs did not seem to show simultaneous expression of SMA with CD34 or p75, some cells expressed CD34^+^ and p75^+^ simultaneously (Fig 1Df and S1Dg-Di Fig)). On day 3, as we have previously reported, SC-re-aggregates became enlarged (to 100–200 ∝m or more in diameter), while some PTMCs attached to the outside of the SC-re-aggregates in a round or elongating form and the rest were localized in a round form in the regions lacking SC-re-aggregates [29] (Fig 2Ga-Ie). It is noteworthy that p75^+^ and CD34^+^ cells formed mixed re-aggregates of various sizes (Fig 2Ga and Gb) in which CD34^+^ cells were distributed throughout the re-aggregates (Fig 2Gc), while p75^+^ cells were located in the outer area of the re-aggregates (Fig 2Gd). In the outer region, some cells expressed p75 and CD34 simultaneously (Fig 2Gf and S1Gg-Gi Fig). When a section was stained with antibodies against p75 and α-SMA, most parts of the re-aggregates were occupied by p75^+^ cells (Fig 2Ha-Hd), though the inner area showed weaker staining for p75 than the outer area, as mentioned above. Most of the α-SMA-positive PTMCs were present in the outermost region of the p-75-positive re-aggregates whose contour line was delineated by alternate chains of PTMCs and p75^+^ cells (Fig 2Ha-He). Some cells appeared to express both antigens simultaneously (Fig 2Hf and S1Hg-Hj Fig). Some PTMCs existed as single or a few cells in areas distant from the p75-positive re-aggregates. When the expression of CD34 and α-SMA was compared in a re-aggregate, areas where two antigens were expressed were almost completely complementary; most parts of the inner and outer areas were occupied by CD34^+^ cells, while only the peripheral area was occupied by PTMCs (Fig 2Ia-Ie). Overlapped regions were rarely observed, but some cells expressed both antigens (Fig 2If and S1Ig-Ij Fig). On day 7 in KSR^+^-medium, seminiferous tubule-like structures are formed, within which most of the SCs align as a single-cell layer at the periphery of the tubule-like structures, with their elongated nuclei oriented with their long axis extending toward the center of the tubules [29] (Fig 2Ja, 2Ka and 2La). The relative positions of CD34^+^ and p75^+^ cells were the same as on day 3 (Fig 2Ja-Jd). Most parts of the contour line of the re-aggregates were delineated by chains of PTMCs, though some parts were occupied by p75^+^ cells (Fig 2Ka and 2Le). Some cells expressed α-SMA and p75 simultaneously (Fig 2Kf and S1Kg-Kj Fig). Also, some cells expressing CD34 and α-SMA were observed (Fig 2Lf and S1Lg-Lj Fig). A one-cell layer (very probably SCs and germ cells) abutted not only the contour lines consisting of α-SMA-positive PTMCs but also those comprising p75^+^ cells (Fig 2Ka, Kb, 2La and Lb). Single or a couple of PTMCs were surrounded by a layer of some cells (probably Sertoli and germ cells) which were assembled in a rosette-like pattern (white arrows in Fig 2La). These results showed that CD34^+^ cells, p75^+^ cells and PTMCs initially existed as re-aggregates (CD34^+^ and p75^+^ cells) or a couple of cells (PTMCs) re-aggregated on their own, and thereafter they mixed together, and then segregated into 3 layers in a re-aggregate in which PTMCs, p75^+^ cells, and CD34^+^ cells were localized in the outermost, middle, and inner layer, respectively. The 3-layer-structure was very similar to the peritubular and interstitial tissues *in vivo*. There also existed some overlapping regions between PTMCs and p75^+^ areas, and between p75^+^ and CD34^+^ areas. These results indicate that the CD34^+^ cells differentiate into p75^+^ cells, which then differentiate into α-SMA-positive PTMCs, and that p75^+^ cells, as well as PTMCs, can support the rearrangement of SCs into a single layer surrounding the interstitial-like and peritubular-like tissue, resulting in the re-construction of seminiferous cord-like and tubule-like structures. Immunostaining for negative controls (without first antibodies) showed no non-specific signals (S2 Fig).

### Proliferation of SCs, CD34^+^ cells, p75^+^ cells and PTMCs during re-aggregate culture

**Fig 3:**
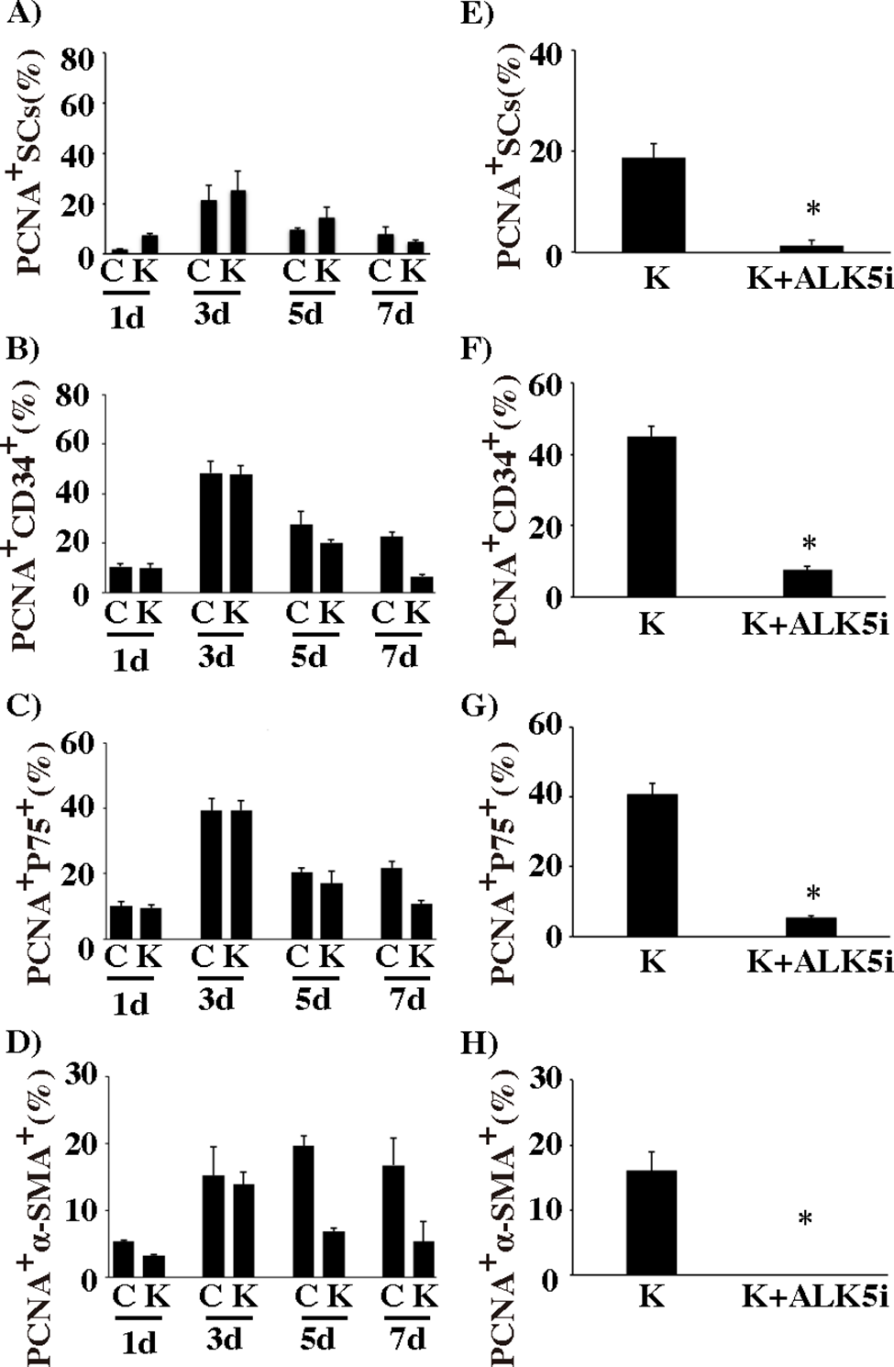
Proliferative activities of SCs, CD34^+^, p75^+^, and α-SMA^+^ cells during culture and its suppression by ALK5i. (A)—(D) The percent of PCNA^+^ SCs, CD34^+^ cells, p75^+^ cells and PTMCs during culture for 7 days in the absence (C) or presence (K) of KSR. (E)—(H) Effect of ALK5i on the percent of PCNA^+^ SCs, CD34^+^ cells, p75^+^ cells and PTMCs after 3 days of culture in the presence of KSR. Pairwise *t*-test with Bonferroni correction was applied to compare values on various culture days in C and K (Raw data, and significant differences were shown in S1 Data). Student’s *t-* test was done between K and K+ALK5i. (*) indicates a significant difference between the two columns. Total numbers of cells examined in 3 experiments were 1500–7000 cells for each day of SCs, and 500–1000 cells for each day of CD34^+^ cells, p75^+^ cells, and α-SMA^+^ cells.

To examine whether the enlargement of re-aggregates consisting of SCs and other types of cells was due to the proliferation of those cells, we examined the proliferative activities of SCs, CD34^+^ cells, p75^+^ cells and PTMCs by assessing PCNA expression during the culture period in the presence and absence of KSR (S3 Fig) (Fig 3). The percent of PCNA^+^ SCs on day 1 was lower than 10%, but the percent rose to the highest value, 20–25%, on day 3, and then decreased to a value lower than 10% by day 7 irrespective of the presence of KSR (Fig 3A). The percent of PCNA^+^ CD34^+^ and p75^+^ cells were highest on day 3 (about 48% and 40%, respectively) with significant differences from those on other days in the presence (K) and absence (C) of KSR (S1 Data), and became lower from day 3 to day 7 (Fig 3B and 3C). It is noteworthy that on day 7 the percent of PCNA^+^ CD34^+^ and p75^+^ cells in the presence of KSR was significantly lower than that in the absence of KSR (S1 Data). This may be due to the fact that re-construction of a tubule structure was completed in the presence of KSR, but not in its absence. The percent of PCNA^+^ α-SMA^+^ cells was highest (about 20%) on day 5 in the absence of KSR (Fig 3D). A significant difference of the percent of PCNA^+^ cells in the presence and absence of KSR was observed on day 5 and 7 (S1 Data). These results indicate that the enlargement of SC-re-aggregates and of CD34^+^-, p-75^+^- and α-SMA^+^-re-aggregates during culture may be partly due to the proliferation of SCs, CD34^+^ cells, p75^+^ cells and PTMCs. As we mentioned in the previous section, some CD34^+^, p75^+^, and α-SMA^+^ cells were observed to co-express one of the other of these antigens.

### Live cell imaging of SCs and other types of cells in re-aggregate cultures shows dynamic processes leading toward re-construction of cord-like and tubule-like structures

**Fig 4:**
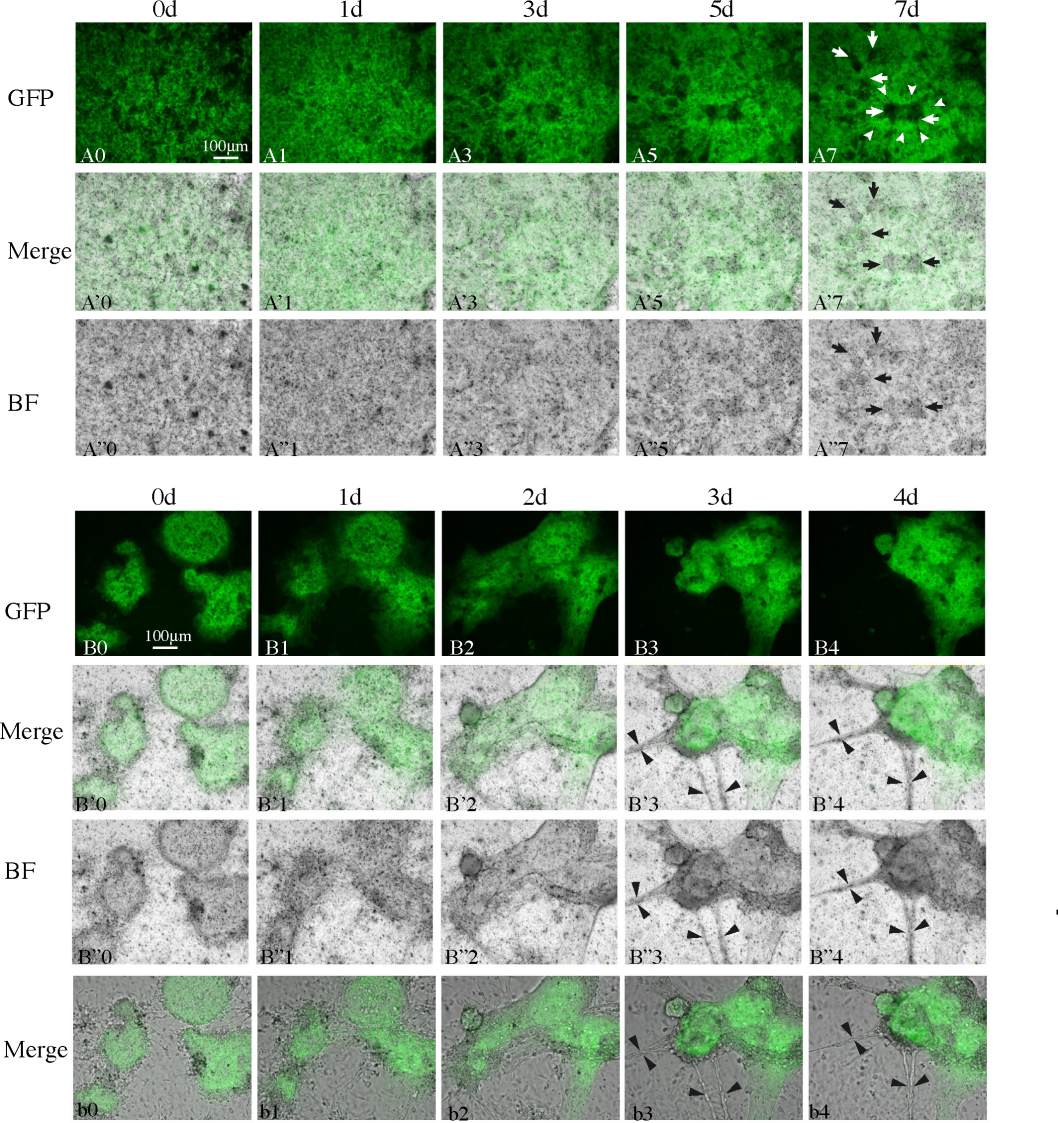
Live cell imaging shows dynamic behavior of SCs and other types of cells during re-aggregate culture. Two typical examples of re**-**aggregate cultures (A0-A”7 and B0-b4) are shown; (A0-A”7) show re-aggregation and segregation of SCs and other types of cells in a large testicular re-aggregate, while (B0-b4) show active movement and coalescence of several small testicular re-aggregates. Fluorescence (GFP) and bright field (BF) images of each culture were recorded, and merged figures are also shown. (b0-b4) show slice snapshots of the same re-aggregate as (B0-B”4). White arrowheads in (A7), and white (A7) and black (A’7 and A”7) arrows show re-aggregates of SCs, and interstitial-like and peritubular-like tissues, respectively. Black arrowheads in (B’3, B’4, B”3, B”4, b3 and b4) show cell-bridges between re-aggregates.

In order to visualize the reconstruction process in re-aggregate cultures, we monitored the behavior of fluorescent SCs in cultures of re-aggregates prepared from *Sox9-EGFP* mice [30]. We also observed other non-fluorescent types of cells in bright-field microscopy simultaneously. When testicular re-aggregates (each > 500 μm in diameter) were observed by time-lapse live cell imaging microscopy in medium containing KSR, the fluorescent SCs and non-fluorescent testicular cells actively moved around and gradually segregated within 2–3 days to form bigger and brighter masses (100–200 ∝m in diameter) of SCs (Fig 4A0-A3) and darker masses of non-fluorescent cells (Fig 4A’0-A’3 (merge), 4A”0-A”3 (BF); S1 Movie (GFP), S2 Movie (BF)), indicating that small SC re-aggregates assembled to form a cord-like structure, while probably CD34^+^ cells, p75^+^ cells and PTMCs (and other cells) congregated to make an interstitial-like structure. Then the segregation processes proceeded further, but with slower speed than before, concomitantly with the expansion in the aggregates’ volume, resulting in the formation of brighter areas consisting of SCs and darker areas consisting of non SCs; the latter were considered to correspond to the interstitial-like tissue (Fig 4A3-A7 (GFP), 4A’3-A’7 (merge), 4A”3-A”7 (BF); S1 Movie (GFP), S2 Movie (BF)). We confirmed by immunostaining that seminiferous cord-like and tubule-like structures were substantially formed in the testicular re-aggregates cultured for 5–7 days in CV-1000 (S4 Fig).

When some small testicular re-aggregates of various sizes (50–500 ∝m in diameter) present on day 0 were observed, they became closer to each other and eventually coalesced into a large re-aggregate within a week (Fig 4B0-B”4). Smaller re-aggregates moved toward, came into contact, and were incorporated into larger re-aggregates, which in turn moved toward and fused with much more larger ones (S3 Movie). Thus, the coalescence of two re-aggregates seemed to be caused by active movement of a smaller one to a larger one. Concomitantly with the coalescence processes, fluorescent SC re-aggregates in each testicular re-aggregate culture became larger in size and brighter, as mentioned above.

When the fluorescent image merged with the corresponding non-fluorescent image was closely observed, occasionally non-fluorescent round cells, and long and slender cells, were observed to have grown out from each testicular re-aggregate (Fig 4B’0-B’4, B”0-B”4, 4b0-b4, S3 Movie). The long slender cells moved toward other re-aggregates and frequently attached with long slender cells that protruded from other re-aggregates at their front tips, forming a bridge between the two re-aggregates. The bridge between the two became thicker by being joined by some other long slender cells (arrowheads in Fig 4B’3, B’4, B”3 and B”4). These figures looked very similar to those in a report by Tung and Fritz [11]. While retaining some bridges, 5 re-aggregates initially present (Fig 4B0, B’0 and B”0) finally fused to become one re-aggregate that was eventually tightly packed (Fig 4B4, B’4 and B”4). Those bridges seemed to have tension, because 2 re-aggregates were pulled by the bridges to fuse into one, or when a re-aggregate was pulled by more than 2 bridges toward various directions, one or 2 of the bridges were occasionally torn off and the re-aggregate was pulled over by other bridges that had stronger force than others (S3 Movie). To observe more clearly the processes of protrusion of cells and bridge formation, a movie of one slice and its snapshot were obtained (S4 Movie; Fig 4b0-b4). To see what cell-types the long slender cells were, we put re-aggregates on collagen-coated coverglasses in a culture chamber, and immunostained those cells which migrated from the re-aggregates with α-SMA, p75 and CD34 antibodies. The results showed that most of the long slender cells were α-SMA-positive PTMCs, though round p75^+^ and CD34^+^ cells also migrated out.

### Interstitial and peritubular cells are required for the formation of seminiferous cord-like and tubule-like structures

**Fig 5:**
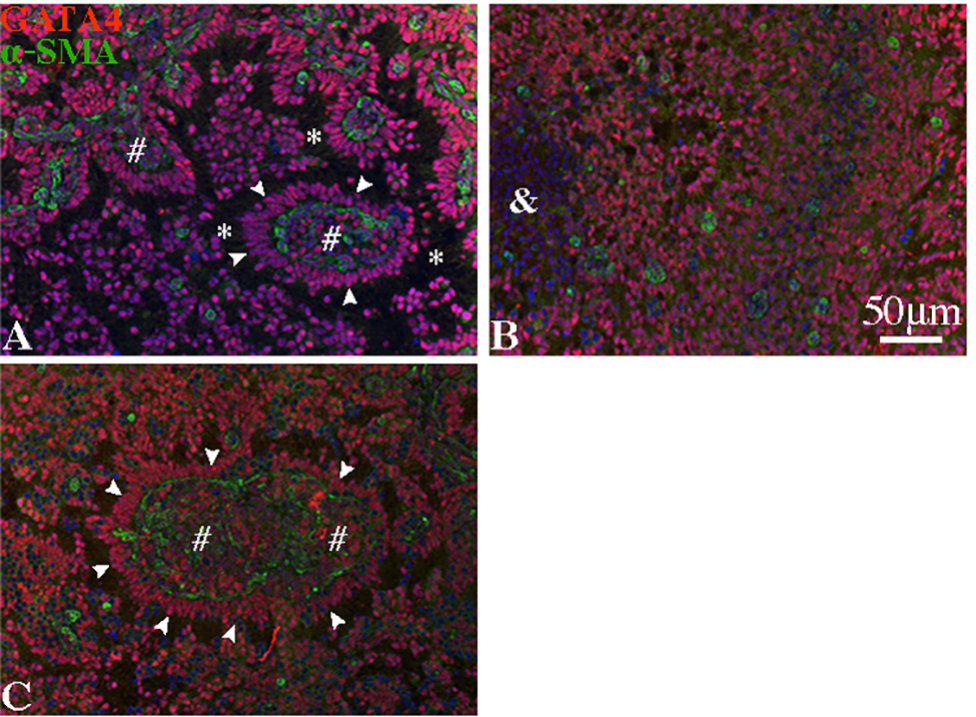
Interstitial and peritubular cells are required for re-construction of cord-like and tubule-like structures. A) Re-aggregate cultures containing all kinds of testicular cells formed normal tubule-like structures after 7 days. B) Removal of interstitial and peritubular cells resulted in dysmorphic structures. C) Re-addition of the interstitial and peritubular cells to the testicular cells that had been deprived of them restored construction of normal tubule-like structures. White arrowheads in (A) and (C) indicate a single layer of SCs. *, #, and & indicate a lumen-like, interstitial-like, and degenerating structure, respectively.

In order to determine whether interstitial and peritubular cells are required for the reconstruction of seminiferous cord-like structures, we performed re-aggregate culture of testicular cells that had been deprived of interstitial and peritubular cells as far as possible. The results showed that SCs were irregularly disposed around a couple of PTMCs that were contaminating in the fraction and it was difficult to distinguish clearly cord-like structures from interstitial-like structures (Fig 5B). When the fractionated interstitial and peritubular cells were added back to the cells that had been deprived of them, however, the reconstructed structures were interstitial tissue-like structures around which there was an orderly arrangement of one layer of SCs with their elongated nuclei protruding into the lumen of tubule-like structures (Fig 5C), which was similar to the structure derived from all the testicular cells (Fig 5A; S5 Fig). These results demonstrate that interstitial and peritubular cells are required for the reconstruction of seminiferous cord-like and tubule-like structures.

### SB431542 and ALK5i inhibit the re-construction of seminiferous cord-like and tubule-like structures

**Fig 6:**
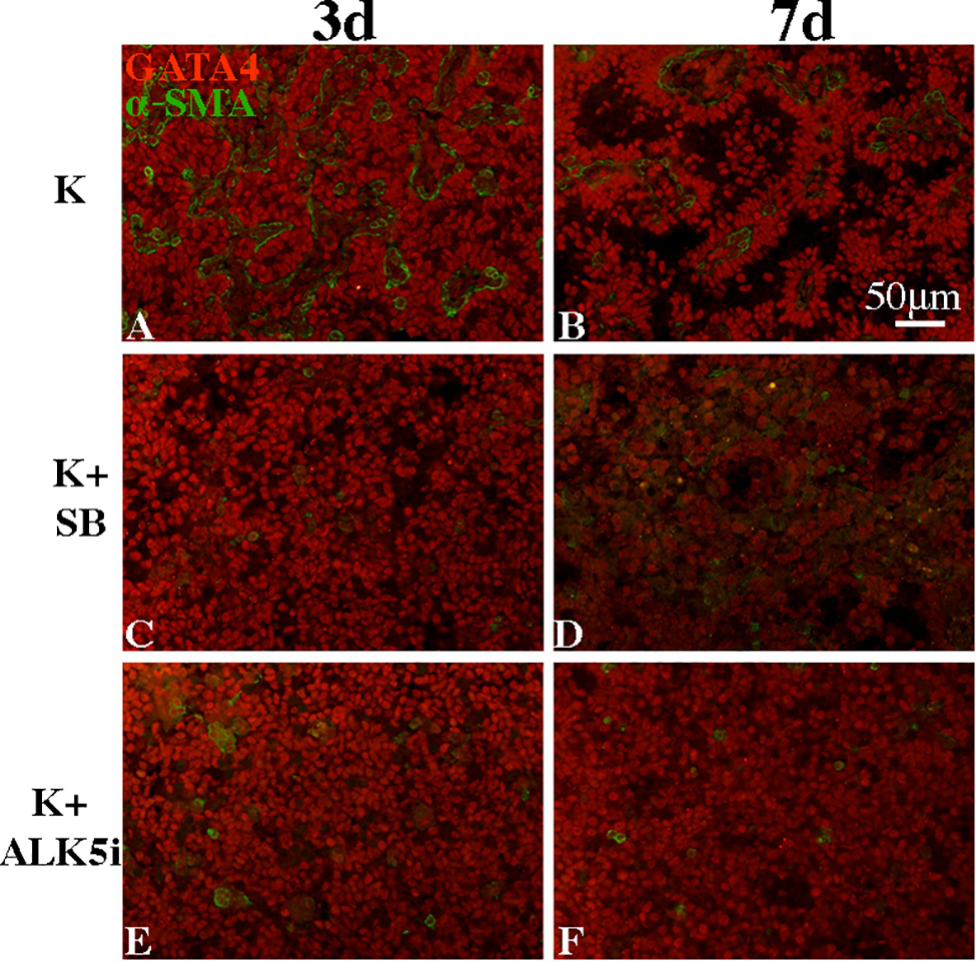
SB431542 and ALK5i disturb the re-construction of seminiferous cord-like and tubule-like structures. Re-aggregates were cultured in medium containing only KSR (A, B), K + 20 ∝M SB 431542 (C, D), or K + 15 ∝M ALK5i (E, F) for 3 (A, C, E) or 7 (B, D, F) days, and immuno-stained with GATA4 (red) and α-SMA (green).

As TGFβ signaling has been shown to be important for testis development [16, 19], we assessed the effects of SB431542 (ALK4/5/7 inhibitor) and ALK5i (ALK5 inhibitor) on the formation of seminiferous cord-like and tubule-like structures in the re-aggregate culture of testicular cells. In KSR^+^-medium without the reagent, the interstitial tissue-like and seminiferous cord-like (and tubule-like) structures were clearly discerned (Fig 6A and 6B, S5 Fig). Ten micromolar SB431542 was not so much effective, compared with 20 ∝M, in disturbing formation of cord-like and tubule-like structures. For ALK5i, 2.5 ∝M and 5 ∝M were not as effective as 15 ∝M. Both SB431542 and Alk5i did not appear to cause cell death. To see if ALK5i caused cell death in re-aggregate cultures, TUNEL staining was performed on samples cultured for 7 days in the absence and presence of ALK5i (2.5 ∝M, 5 ∝M and 15 ∝M) (S6 Fig). The results in the absence of ALK5i showed that dead cells among 8115 cells (total) in 11 sections in 3 experiments was 2.06 ± 0.01%. On the other hand, in the presence of 2.5 ∝M, 5 ∝M and 15 ∝M ALK5i, dead cells were 3.7 ± 0.08%, 4.0 ± 0.77%, and 3.8 ± 0.39% among 5384 cells (7 sections), 6183 cells in (8 sections), and 10210 cells (11 sections), respectively, in 3 experiments. Thus, the percentages of dead cells in different concentrations of ALK5i were almost constant. Incubation with 20 ∝M SB431542 from day 0 to 3 and to 7 resulted in a lack of seminiferous cord-like and tubule-like structures, respectively; instead, the distinction between the interstitial tissue-like and seminiferous cord-like (and tubule-like) areas became unclear; the nuclei of SCs were round and irregularly arranged, and most of the PTMCs were round and randomly distributed (Fig 6C and 6D). ALK5i (15 ∝M) had the same effect as SB431542 (Fig 6E and 6F). These results indicate that TGFβ signaling through ALK5 is indispensable for the reconstruction of cord-like and tubule-like structures.

### ALK5i interferes with the re-construction of interstitial-like and peritubular-like tissues by CD34^+^, p75^+^ and PTMCs

**Fig 7:**
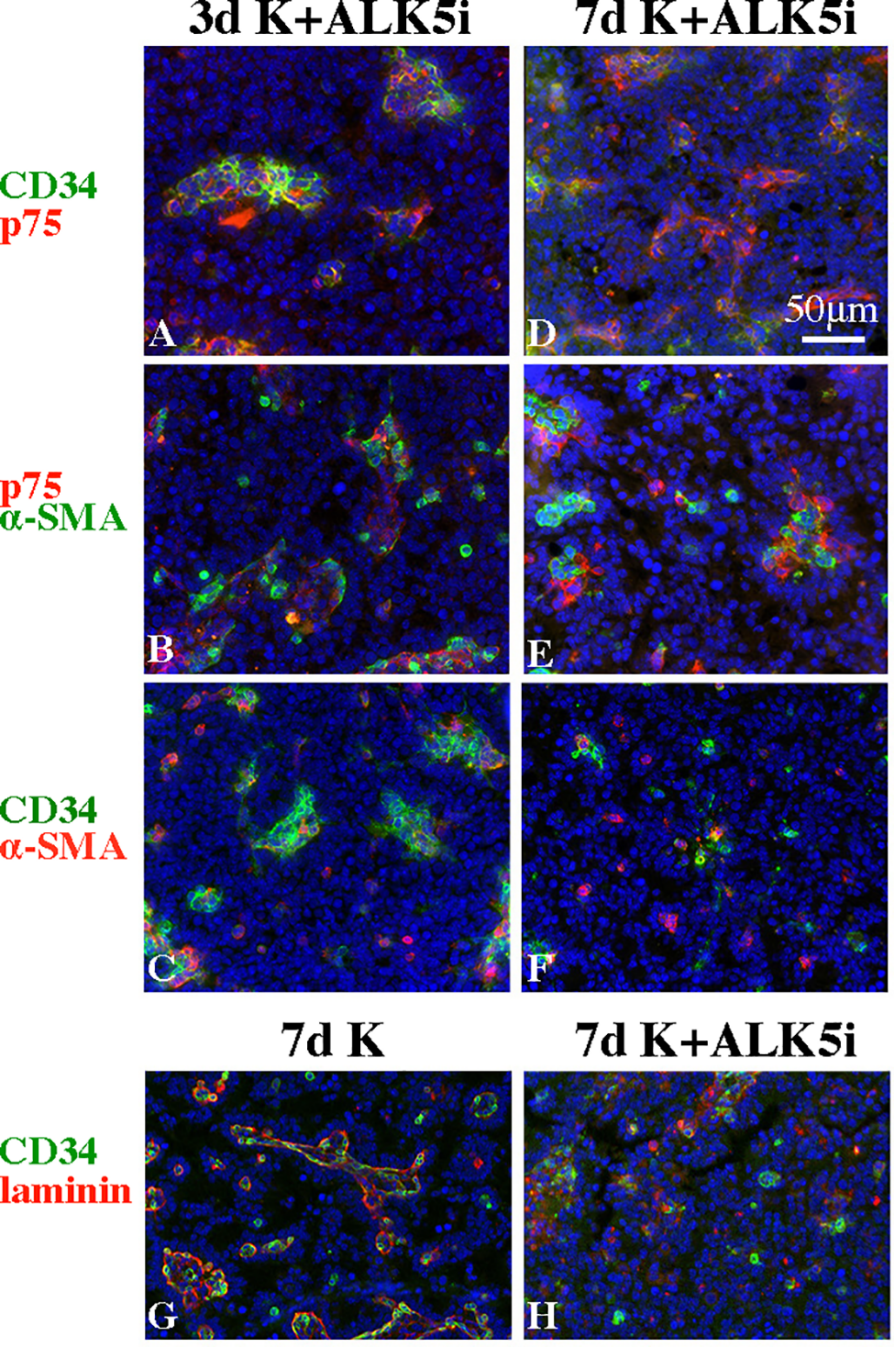
ALK5i interferes with the re-construction of interstitial-like and peritubular-like tissues by CD34^+^, p75^+^ and PTMCs. Behaviors of CD34^+^, p75^+^ cells, and α-SMA^+^ cells were assessed after culture of testicular re-aggregates for 3 (A, B, C) or 7 days (D, E, F) in the presence of K+ALK5i. Laminin expression was also disturbed by incubation with ALK5i (H) for 7 days, compared with that without ALK5i (G).

Since we have shown that interstitial and peritubular cells are required for the reconstruction of seminiferous cord-like structures, we assessed the effect of ALK5i on the behavior of CD34^+^ cells, p75^+^ cells and PTMCs during re-aggregate culture of testicular cells. When ALK5i was added to the cultures for 3 days, most of the re-aggregates of CD34^+^ and p75^+^ cells decreased in size to < 50 ∝m in diameter (Fig 7A, 7B and 7C), and in the re-aggregates CD34^+^ and p75^+^ cells were not clearly localized into the inner and outer regions, respectively. PTMCs were localized not only in the outermost region, but also in the inner region of the re-aggregates of p75^+^ or CD34^+^ cells (Fig 7B and 7C). When Alk5i was added for 7 days, the size of re-aggregates consisting of CD34^+^ and p75^+^ cells was smaller than that of re-aggregates cultured without the reagent, and these two types of cells did not necessarily co-exist in the same re-aggregates (Fig 7D), in contrast to the re-aggregates formed in medium without the reagent, most of which contained the two types of cells (Fig 1J). Also, PTMCs were observed to exist as randomly mixed re-aggregates with p75^+^ cells (Fig 7E) or CD34^+^ cells (Fig 7F). These results indicate that ALK5i disturbed the interactions among CD34^+^ cells, p75^+^ cells and PTMCs, and the formation of orderly structures.

We investigated the effect of ALK5i on the laminin expression pattern in the re-aggregates, since laminin is produced by SCs, and its production is increased by the interaction of SCs with PTMCs *in vitro* [12]. Laminin has been shown to be important for the formation of cord-like structures *in vitro* [13]. Laminin expressed in KSR^+^ medium delineated the contour lines of each re-aggregate that consisted of CD34^+^ or p75^+^ cells with an outermost region that was occupied by PTMCs (Fig 7G), whereas it appeared as spots in ALK5i-containing medium after culturing for 7 days (Fig 7H). These results indicate that Alk5i disturbed the interactions between PTMCs and SCs necessary for laminin production and/or deposition.

### ALK5i suppresses the proliferation of SCs, CD34^+^, p75^+^ and PTMCs in re-aggregate culture

Next we examined whether ALK5i suppresses the proliferation of SCs, CD34^+^ cells, p75^+^ cells or PTMCs. Incubation with ALK5i for 3 days in KSR^+^-medium decreased the percent of PCNA^+^ SCs” (Fig 3E). ALK5i also suppressed remarkably the percent of PCNA^+^ CD34^+^ cells, p75^+^ cells and PTMCs (Fig 3F–3H). These results indicate that ALK5 signaling is involved in the proliferation of not only SCs but also CD34^+^ cells, p75^+^ cells and PTMCs.

### Live cell imaging showed that ALK5i compromises motility of SCs and other types of cells in re-aggregate culture

**Fig 8:**
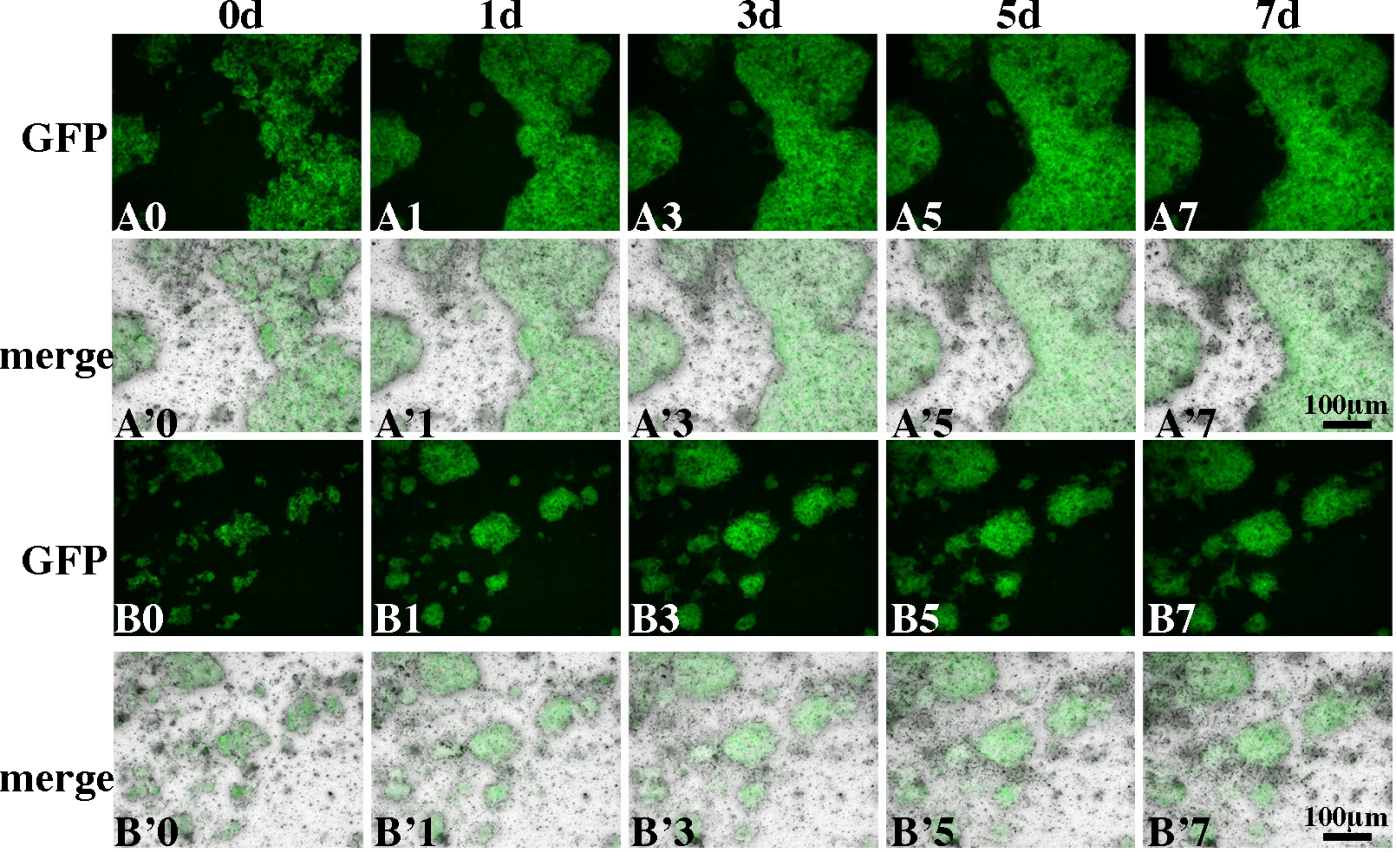
ALK5i suppresses motility of SCs and other types of cells. Two typical examples of re-aggregate cultures (A0-A’7 and B0-B’7) are shown; (A0-A’7) show culture of a large re-aggregate in which segregation of SCs and other types of cells was slowed, while (B0-B’7) show lack of coalescence of several small testicular re-aggregates. Snapshots recorded as fluorescent images (A0-A7, B0-B7) and merged with bright field images (A’0-A’7, B’0-B’7) are shown.

Next, in order to elucidate how ALK5i affects the re-construction of cord-like and tubule-like structures, we observed the behavior of SCs and other types of cells by live cell imaging. In the presence of ALK5i the fluorescing SC re-aggregates became larger in size to some extent and slightly brighter after a few days, forming re-aggregates less than about 40 ∝m in diameter (Fig 8A0-A3; Fig 8A’0-A’3). However, the SC-re-aggregates did not expand further to more than 50–60 ∝m in diameter within a week (Fig 8A3-A7; Fig 8A’3-A’7). It was noteworthy that the motion of most testicular cells was clearly slower than that of those with KSR only. In contrast to the cultures in KSR^+^-medium, small testicular re-aggregates barely moved, and rarely fused with each other to form larger re-aggregates (Fig 8B0-B7; Fig 8B’0-B’7). While in the absence of ALK5i spear-like cells frequently shortened and elongated actively (S4 Movie), those cells could not elongate again once they shortened in length in the presence of ALK5i. Thus formation of bridges among the re-aggregates did not occur, and therefore the re-aggregates did not fuse. These results indicate that TGFβ signaling through ALK5 is involved in the motility of SCs, and other types of cells, probably CD34^+^, p75^+^ and PTMCs, and thus in the re-construction of cord-like and tubule-like structures.

### Culture of purified CD34^+^ cells demonstrated that CD34^+^ cells can differentiate into p75^+^ cells, which can proceed to PTMCs, and the differentiation is compromised by ALK5i

**Fig 9:**
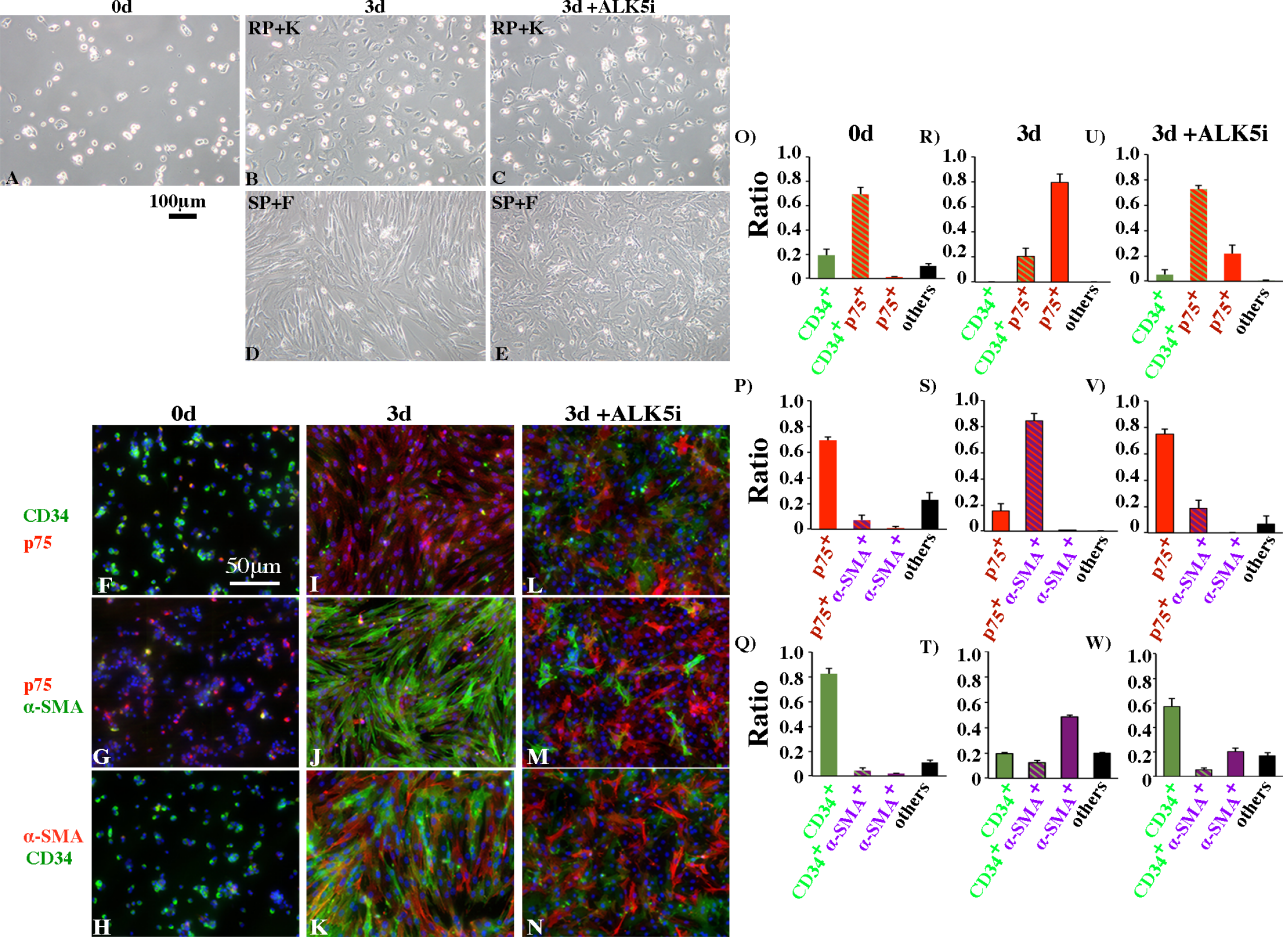
CD34^+^ cells differentiate into p75^+^ cells, and then to PTMCs, and ALK5i compromises the differentiation. (A)~(E) Phase-contrast photomicrographs of purified CD34^+^ cells cultured on coverglasses for 0 day (A), 3 days in RP+K medium (B), in the same medium as (B) but with ALK5i (C), in SP+f medium (D), or in the same medium as (D) but with ALK5i (E). (F)-(N) Expressions of CD34, p75 and α-SMA were assessed by immunofluorescence on day 0 (F-H), day 3 in SP+f medium (I-K), or day 3 in the same medium as (I-K) but with ALK5i (L-N). (O)-(W) Percentages of cells expressing CD34, p75, α-SMA, two of the three, or none were calculated for each combination of two kinds of antibodies among the three on day 0 (O-Q), day 3 in SP+f (R-T), and day 3 in the same medium but with ALK5i (U-W). Total numbers of cells examined in 3 experiments were 500–1000 cells for each combination of 2 antibodies among 3 (CD34, p75, and α-SMA) on each day in the presence and absence of ALK5i.

To assess whether CD34^+^ cells can differentiate into p75^+^ cells, and p75^+^ cells can proceed to differentiate into α-SMA-positive PTMCs, purified CD34^+^ cells were cultured on coverglasses coated with collagen. A few hrs after inoculation at 34°C, most of the cells attached to the coverglasses, and some of them started to extend their cytoplasm (Fig 9A). To examine the purity of the cells, cells attached to coverglasses were immunostained with pairs of three antibodies (CD34, p75, and α-SMA). When examined with anti-CD34 and -p75 antibodies, the percentage of cells stained with CD34 only was about 20%, while about 70% co-expressed CD34 and p75 simultaneously (Fig 9F and 9O; S7 Fig). Percentage of cells that were not stained with either antibody was about 10%. When the cells on a coverglass were stained with anti-p75 and -α-SMA antibodies, about 70% were positive only for p75, while only about 5% were double-positive for p75 and α-SMA (Fig 9G and 9P). The rest were not stained by either antibody; these cells were thought to include cells positive for CD34^+^ only. When the cells on a coverglass were examined for expression of CD34 and α-SMA, about 82% were positive for CD34 only (Fig 9H and 9Q), indicating that these cells included cells double-positive for p75. Only about 4% of cells were double-positive for CD34 and α-SMA. These results indicate that purity of the CD34^+^ cells was about 90%, while about 78% of the total CD34^+^ cells were double-positive for p75. Few cells were positive for α-SMA alone and about 7% of cells were double-positive for p75 and α-SMA.

Two kinds of media were used for culture; one was RPMI-1640 containing 10% KSR (RP + K medium), and the other was StemPro/RPMI-1640 containing 20% fbs (SP +f medium), based on a previous report which showed it is suitable for culture of CD34^+^ stromal cells [31].

On day 3 the cell shape in RP + K medium was fibroblastic (Fig 9B), whereas the cells cultured in SP + f medium were long and slender spindle-shaped (Fig 9D). To examine whether CD34^+^ cells differentiate into p75^+^ cells, and the latter differentiate into α-SMA^+^ cells, an immuofluorescence study was performed. When the cells cultured in SP+f medium for 3 days were stained with CD34 and p75 antibodies, CD34^+^ only cells decreased to none, and cells double-positive for CD34 and p75 also decreased to about 20% (Fig 9I and 9R). In contrast, p75^+^ only cells robustly increased to about 80%. When the cells were stained with p75 and α-SMA, cells double-positive for p75 and α-SMA increased to about 84%, while p75^+^ only cells decreased to about 15% (Fig 9J and 9S). When the cells were examined for CD34 and α-SMA expressions, SMA^+^ only cells accounted for about 48% (Fig 9K and 9T). Cells positive for CD34 only accounted for about 20%, and cells positive for CD34 and α-SMA accounted for about 12%. Also, there were cells not stained by either antibody. The results for cells cultured in RP+K medium were also similar to those cultured in SP+f medium, though the differentiation capacity was better in SP+f (data not shown). These results indicate that CD34^+^ cells differentiated into p75^+^ cells, which then differentiated into α-SMA-positive PTMCs. It is also possible that CD34^+^ cells may differentiate into α-SMA-positive PTMCs directly.

Then we investigated the effect of ALK5i on the differentiation of CD34^+^ cells into p75^+^ cells and PTMCs. When ALK5i was added to RP+K medium, the cells cultured in the medium were thin and sometimes stellate with long cytoplasmic processes (Fig 9C). In SP+f medium, cells that would have shown a long, slender shape oriented in the same direction in the absence of ALK5i (Fig 9D) changed into shortened and irregular shaped cells (Fig 9E). When cells were immunostained with antibodies for CD34 and p75, the percentage of cells expressing p75 alone decreased to about 22%, whereas cells double-positive for CD34 and p75 increased drastically to about 73% (Fig 9L and 9U). When the expressions of p75 and α-SMA were examined, the number of double-positive cells decreased to about 20%, whereas cells positive only for p75 increased robustly to about 75% (Fig 9M and 9V). These cells are considered to include CD34^+^ cells. When CD34/α-SMA expressions were assessed, cells positive only for α-SMA decreased significantly to about 20% (Fig 9N and 9W). In contrast, cells positive only for CD34 increased remarkably to about 57%. These results indicate that ALK5i compromised the differentiation of CD34^+^ cells into p75^+^ cells, and into PTMCs by direct action on CD34^+^ cells and also on p75^+^ cells.

## Discussion

Our current study revealed various findings about the re-construction of cord-like and tubule-like structures in re-aggregate cultures of testicular cells as follows: 1) Ordered structures similar to peritubular and interstitial tissues *in vivo* were reconstructed in re-aggregates consisting of PTMCs, p75^+^ and CD34^+^ cells. 2) Interstitial and peritubular cells were required for re-construction of cord-like and tubule-like structures. 3) Live cell imaging showed that dynamic cell movement and segregation occurred during the re-construction of cord-like and tubule-like structures. 4) ALK5i disturbed the proliferation of CD34^+^ cells, p75^+^ cells, PTMCs, and SCs, as well as movement of SCs and other types of cells including peritubular cells. 5) ALK5i compromised the re-construction of interstitial-like and peritubular-like structures and cord-like and tubule-like structures. 6) Purified CD34^+^ cells cultured on coverglasses differentiated into p75^+^ cells and PTMCs, and ALK5i suppressed this differentiation.

During the re-aggregate culture, CD34^+^ and p75^+^ cells that initially existed as re-aggregates on their own, and PTMCs that were distributed mostly as single cells became intermingled with each other, and concomitantly with proliferation formed re-aggregates within 3 days consisting of 3 layers; the inner layer was CD34^+^ cells, the outer one was p75^+^ cells, and the outermost one, namely the contour line of the re-aggregates, was PTMCs around which cord-like structures made by SCs formed. It is noteworthy that the 3-layer structures consisting of CD34^+^ cells, p75^+^ cells and PTMCs are similar to the distribution of those cells in prenatal and neonatal testes. Previous studies have shown that p75^+^ cells occupy the whole intertubular compartment around testis cords in murine prenatal testes, and progressively concentrate around the testis cords [4]. The high expression of p75 as a layer around a PTMC layer that surrounds each testis cord decreases and disappears at puberty [4, 32]. Kuroda et al. [33] showed CD34^+^ stromal cells are distributed in the outer layer of the PTMC layer in the peritubular area, as well as in the interstitial tissue in fetal, neonatal and adult human testes. Thus, these 3-layer structures abutting the SC-layer seemed to mimic the interstitial and peritubular tissue and contained most of the LCs present in the testicular re-aggregates [29], and hence it is considered that interstitial-like and peritubular-like tissues of the testis as well as of the cord-like and tubule-like structures were successfully re-constructed.

In our current study, some CD34^+^ cells were found to express p75 simultaneously on day 0 of culture, and some p75^+^ cells expressed α-SMA simultaneously during the re-aggregate culture, indicating that CD34^+^ cells differentiate into p75^+^ cells, which then differentiate into PTMCs. Our present results about cultures of purified CD34^+^ cells also showed that some of them co-expressed P75. Some p75^+^ cells were also shown to co-express α-SMA during cell culture. The transition of a CD34^+^ cell-rich population into p75^+^ cell-rich and into α-SMA-rich populations when cultured in StemPro + fbs medium, as well as in RPMI + KSR, demonstrates that CD34^+^ cells are able to differentiate into p75^+^ cells, and the latter have the capacity to differentiate into PTMCs. These results strongly indicate that CD34^+^ cells can be precursors of PTMCs by way of p75^+^ cells in the *in vivo* testis. In cell cultures of CD34^+^ cells, however, about 30% of the total cells co-expressed CD34 and α-SMA after 3 days. This raises two possibilities; one is that CD34^+^ cells differentiate into α-SMA^+^ cells by way of cells that express three kinds of antigens simultaneously, and the other is that CD34^+^ cells can differentiate into α-SMA^+^ cells directly as well as by way of p75^+^ cells. These results are consistent with those by Campagnolo et al. [32] who showed that p75^+^ cells are able to differentiate into PTMCs *in vitro*, indicating that the former are the precursors of the latter. In addition, Jeanes et al. [34] showed that p75^+^ cells also express PECAM-1, an endothelial cell marker, in mouse embryonic testis. Thus, it seems reasonable to suggest that *in vivo* CD34^+^ endothelial cells are precursors of p75^+^ cells, which then differentiate into PTMCs. CD34 is expressed in various kinds of cell types, such as hematopoietic stem and progenitor cells, muscle satellite cells, interstitial cells, and vascular endothelial progenitors [35]. Although whether it has a common function in these various cell types remains to be determined, CD34 is regarded as a general marker of progenitor cells. Cell lineage analysis of CD34^+^ cells in prenatal and postnatal testis remains to be performed, but should give insight into this question.

Then how are these orderly re-aggregates corresponding to interstitial-like and peritubular-like tissues re-constructed? Distinctive behaviors of PTMCs, p75^+^ and CD34^+^ cells observed during culture offer some indications about their roles in re-construction of interstitial-like and peritubular-like tissues. On day 3 the inner layer of the re-aggregates of p75^+^ and CD34^+^ cells consisted mainly of CD34^+^ cells and the outer layer comprised p75^+^ cells, while some CD34^+^ cells located in the outer region co-expressed p75. This indicates that CD34^+^ and p75^+^ cells first re-aggregate with each other, and then the two types of cells segregate into the inner and outer parts, respectively, while CD34^+^ cells located in the outer region differentiate into p75^+^ cells. Proliferation of both cell types is considered to result in enlargement of the re-aggregates. On the other hand, on day 3 some PTMCs attached to the surface of the outer layer that consisted mainly of p75^+^ cells, while the rest were distributed solely as single cells, apart from the re-aggregates of CD34^+^ and p75^+^ cells. The outermost layer, namely the contour line of the re-aggregates, comprised PTMCs and p75^+^ cells that were connected alternately. Some cells positive for p75 on day 3–7 were found to be positive simultaneously for α-SMA. These results indicate two possibilities: PTMCs situated at the outermost line of the re-aggregates were supplied by migration and attachment of those that had been interspersed on day 0 and/or by differentiation of p75^+^ cells situated in the outer region into PTMCs.

As for the role of PTMCs in testes formation, many investigators have reported interactions between SCs and PTMCs *in vitro*. SCs and PTMCs in co-culture co-operatively form and deposit ECM components such as laminin [12]. The ECM has been reported to be important in testicular cord formation, since SCs cultured within reconstituted basement membrane gels undergo a morphogenesis into a structure which resembles cords, though the contributions of contaminating PTMCs to the structures are unknown [10]. When SCs and PTMCs are co-cultured on plastic culture dishes, SCs form mounds or nodules surrounded by PTMCs [9]. Since basement membrane was observed between SCs and PTMCs, the nodules were thought to resemble some aspects of the seminiferous tubules, though no polar SCs or lumen were observed within the nodules. In our current study, re-aggregates consisting of several tens or hundreds of PTMCs only were barely observed after culture for 3–7 days, but dispersed single or pairs of PTMCs were occasionally observed besides the constituent cells at the outermost layer of re-aggregates within which p75^+^ and CD34^+^ cells formed the outer and inner layers, respectively. Around these single or pairs of PTMCs some SCs were found to assemble radially like a rosette, but those PTMCs did not seem to have a capacity to create a cord-like or tubule-like structure with normal size. Also, when interstitial and peritubular cells were depleted, re-aggregate cultures of cells mainly consisting of SCs and germ cells failed in re-construction of cord-like structures, but formed rosette-like radial assemblies of a small number of SCs around a couple of PTMCs which were not depleted from the preparation (Fig 4B). On the other hand, p75^+^ and CD34^+^ cells were able to make re-aggregates comprising several tens to hundreds of cells on their own or together. SCs aligned as one layer around re-aggregates of p75^+^ and CD34^+^ cells of which the outermost layer consisted of PTMCs and p75^+^ cells alternately. This indicates that p75^+^ cells as well as PTMCs situated at the outermost layer of the re-aggregates are able to interact with abutting SCs to help organize them to form a single layer of SCs with a polarity of the basal (proximal) to distal axis, and secrete laminin to make a basement membrane.

Live cell imaging is a strong tool to analyze dynamic behavior of various cell-types labeled depending on a study’s purpose [36, 37, 38]. Nel-Themaat et al. [39] used *Sox9-EGFP* knock-in mice to analyze SC behavior in the intact embryonic testis and discovered that testis cord formation is initially highly dynamic in that SCs are readily exchangeable between neighboring cords. SCs initially have mesenchymal morphologies and behaviors, but later, the cells become epithelialized in testis cords [40]. Our current study using *Sox9-EGFP* mice has revealed that active and dynamic movement of SCs and other types of cells was involved in the re-construction of cord-like and tubule-like structures. Observation of testicular re-aggregates in medium containing KSR indicated that the processes leading toward formation of tubule-like structures could be divided into an earlier (day 0–3) and a later (day 3–7) period. During the earlier period, the fluorescent SCs and non-fluorescent testicular cells actively moved around and gradually segregated to form bigger and brighter masses of SCs and darker masses of non-fluorescent cells. It seems that small SC re-aggregates assembled to form a cord-like structure, while non-fluorescent cells, probably including CD34^+^ cells, p75^+^ cells and PTMCs, congregated to make an interstitial-like structure. During the later period the cord-like area consisting mainly of SCs and interstitial-like area expanded its volume, while the segregation processes proceeded further but with slower speed than in the earlier period, resulting in the formation of tubule-like structures. Observation of some small testicular re-aggregates of various sizes on day 0 revealed intriguing behaviors of re-aggregates and the cells coming out of the re-aggregates. Occasionally, long, slender cells coming out of the re-aggregates moved toward other re-aggregates and frequently attached with those that protruded from other re-aggregates at their front tips, forming a bridge between the two re-aggregates. These phenomena seem to be similar to the observations by Tung and Fritz [11] in which PTMCs migrated out from SC-enriched aggregates plated on type I collagen and oriented themselves in a direction pointing toward adjacent aggregates. Although coalescence of two adjacent aggregates was not described in their report, we observed the coalescence of some re-aggregates that we considered to be caused by the tension generated by those cell-bridges consisting mainly of PTMCs and p75^+^ cells. Also, some cells, probably PTMCs, were frequently observed to elongate and shorten outside and inside re-aggregates by live cell imaging. This indicates that PTMCs are able to change their shape from round to elongated, and vice versa, and thus flexibly contribute to the formation of cord-like and tubule-like structures. It seems likely that these flexible abilities of cell shape change by PTMCs (and probably some p75^+^ cells) and tension changes exerted by chains of PTMCs and p75^+^ cells play a pivotal role in the re-construction of cord-like and tubule-like structures.

Our results indicated that ALK5i inhibited the proliferative activities of CD34^+^ and p75^+^ cells, and PTMCs, as well as SCs, in re-aggregate cultures. SCs are known to proliferate during fetal and early neonatal life in rodents, and activin A, a member of the TGFβ superfamily, is produced in LCs and acts directly on SCs to promote their proliferation through SMADs [41, 42]. However, SC proliferation was also suppressed by ALK5i, indicating that both activin and TGFβ signaling contribute to promotion of SC proliferation during testis formation [19]. ALK4/ALK7 Signals by TGFβs, as well as activins and nodal, are transduced through activation of SMAD2 and/or SMAD3 [43, 44]. Interestingly, SMAD3 is localized in the nuclei of interstitial cells and PTMCs, as well as SCs, in postnatal mouse testes [44]. Collectively, these and our results indicate that there may be multiple TGFβ superfamily ligands other than activin A, which stimulate the proliferation of SCs, and also CD34^+^ and p75^+^ cells, and PTMCs.

ALK5i also disturbed the reconstruction of cord-like and tubule-like structures, making the distinction between cord-like and interstitial-like areas obscure. ALK5i not only disrupted the orderly arrangement of SCs but also compromised the 3-layer structure of the interstitial-like area, which would consist of PTMCs, p75^+^ cells and CD34^+^ cells from the outermost to the innermost region, in the absence of ALK5i. Consistently, ALK5i disrupted the deposition of laminin in between SCs and PTMCs. In addition, live cell imaging showed that ALK5i suppressed the motility of the cells that was observed during the reconstruction of cord-like and tubule-like structures. Thus, these results indicate that ALK5 signaling is involved in many aspects of cellular activities during the reconstruction of cord-like and tubule-like structures. Miles et al. [19] also showed that TGFβ signaling through ALK5 is required for testis cord formation from E11.5-E12.5 gonad in organ culture. How does ALK5i compromise the reconstruction of cord-like and tubule-like structures? Treatment of purified CD34^+^ cells in culture with ALK5i not only drastically affected the cell shape but also blocked the cells’ differentiation into p75^+^ cells and PTMCs, indicating that ALK5i directly affected CD34^+^ cells and p75^+^ cells. Considering all these results, we suggest that ALK5 signaling is indispensable for reconstruction of ordered and functional cord-like and tubule-like structures, as well as of interstitial-like and peritubular-like structures, through acting primarily on CD34^+^ and p75^+^ cells (and PTMCs). On the other hand, live cell imaging showed that ALK5i impeded the movement and segregation of both SCs and other types of cells (CD34^+^ and p75^+^ cells and PTMCs). SCs are known to be major regulators of testicular morphogenesis, and mutual interactions between SCs and various cell-types in testes, including PTMCs and LCs, are indispensable for their differentiation [1, 2, 3, 45, 46]. Thus it is also possible that ALK5i affects CD34^+^ and p75^+^ cells and PTMCs through acting on SCs, or both.

Thus, we showed that re-construction of interstitial-like and peritubular-like tissues was successful in 3D-reaggregate culture of neonatal testicular cells, and that interstitial and peritubular cells were indispensable for re-construction of cord-like and tubule-like structures. CD34^+^ endothelial cells are considered to play a pivotal role as progenitor cells of p75^+^ cells and PTMCs. A critical question is how well this *in vitro* re-construction system recapitulates the mechanisms underlying cord formation *in vivo*. Cell migration from the mesonephros to gonad in embryonic testis is required for the development of cord and interstitial compartments, and some previous studies suggested that the migrating population includes PTMCs, endothelial cells, and other interstitial cells [6, 7, 8]. On the other hand, Cool et al. [14] and Combes et al. [15] showed that immigrant cells are not PTMCs, or other interstitial cells, and that migrating endothelial cells play an important role in the formation of the testis cords by formation of the vascular network of the testis. Whether vascularization occurs in our culture system remains to be clarified. Future studies using *in vitro* testis re-construction culture systems will contribute to addressing many important questions on the mechanisms underlying the establishment and/or maintenance of testis cord and tubule structures, such as how germ cells and LCs are sorted on the inside and outside of seminiferous cord structures, respectively; how cords develop into tubules; and whether spermatogonial stem cell niches are formed, and if so how?

## Supporting information

S1 Fig Immunostaining shows co-expression of two antigens among CD34, p75 and α-SMA. Merged figures (Ag and Dg) with DAPI in the same area as those in Aa and Da (areas encircled by white dotted lines), respectively, in Fig 1 and their resolved figures into CD34 (Ah and Dh) and p75 (Ai and Di) with DAPI were shown. Also merged figures (Gg, Hg, Ig, Jg, Kg, and Lg) with DAPI in the same area as those encircled by white dotted lines in Ga, Ha, Ia, Ja, Ka, and La, respectively, in Fig 2 and their resolved figures into CD34, p75 and α-SMA with DAPI were shown in each row. White arrowheads show cells that expressed two antigens simultaneously.
(PDF)

S2 Fig Negative controls for antibodies used in Figs 1 and 2, S1 Fig and S3 Fig. A) A’) D) D’) Negative controls without first antibody against CD34 were shown in A (with DAPI), A’ (without DAPI), D (with DAPI) and D’ (without DAPI). A and A’ were stained only by the secondary antibody, A21208, and D and D’ were stained only by the secondary antibody, ab150073. B) B’) Negative controls for p75 antibody stained by only secondary antibody, ab150076. C) C’) E) E’) Negative controls for α-SMA antibody stained by only secondary antibodies, ab150105 and ab150108. F) F’) Negative controls for GATA4 antibody stained by only secondary antibody, ab150132.
(PDF)

S3 Fig Immunostaining shows PCNA-positive SCs, CD34^+^ cells, p75^+^ cells and α-SMA^+^ PTMCs. (A)~(D) Double immunostaining with antibodies against PCNA (green) and GATA-4 (red), and antibodies against PCNA (red) and CD34 (green), p75 (green), or α-SMA (green) in the presence of KSR on day 3 of culture. (E)~(H) Double immunostaining with antibodies against PCNA (green) and GATA-4 (red) (E), and antibodies against PCNA (red) and CD34 (green) (F), p75 (green) (G), or α-SMA (green) (H) in the presence of KSR + ALK5i on day 3 of culture.
(PDF)

S4 Fig Seminiferous tubule-like structures were reconstructed after time-lapse recording in CV1000. A) Cultured for 5 days. B) Cultured for 7 days.
(PDF)

S5 Fig Magnified view of lumen structures. (A) and (C) Double immunostaining of sections cultured for 7 days in the presence of KSR with antibodies against GATA-4 (red) and laminin (green) (A) or α-SMA (green) (C). (B) and (D) Same sections as (A) and (C), respectively, stained with DAPI. (*) shows lumen structures.
(PDF)

S6 Fig TUNEL staining of re-aggregate cultures. Sections from re-aggregates cultured for 7 days in the absence (A) and presence (B) of 15 ∝M ALK5i were stained with TUNEL and methyl green.
(PDF)

S7 Fig Overlapping expression of CD34 and p75, p75 and α-SMA, α-SMA and CD34 in cultured cells. Cells showing both CD34 and p75 (Aa ~ Ac), those double-positive for p75 and α-SMA (Ba~Bd), and those expressing both CD34 and α-SMA are indicated by white arrows.
(PDF)

S1 Movie Time-lapse recording of the behavior of fluorescent SCs in cultures of re-aggregates prepared from *Sox9-EGFP* mice. (ZIP)

S2 Movie The same sample as in S1 Movie was recorded simultaneously by bright field microscopy. (ZIP)

S3 Movie Time-lapse recording of the behavior of SCs and other types of cells in cultures of small testicular re-aggregates, and shown in merged figures taken by fluorescence microscopy and those by bright field microscopy. (ZIP)

S4 Movie Time-lapse recording of the behavior of SCs and other types of cells of the same sample as in S3 Movie, but only one slice was recorded. (ZIP)

S1 Data A) Raw data and their comparisons of the percentage of PCNA^+^ SCs, CD34^+^ cells, p75^+^ cells and PTMCs during culture for 1, 3, 5, and 7 days in the absence (C) and presence (K) of KSR. Pairwise comparisons were carried out by *t*-test with Bonferroni correction. B) The solid lines indicate significant differences (p < 0.05).
(XLSX)
