# Supplementary figures and images for: Roles of CD34+ cells and ALK5 signaling in the reconstruction of seminiferous tubule-like structures in 3-D re-aggregate culture of dissociated cells from neonatal mouse testes
Source: PLoS One. 2017 Nov 30;12(11):e0188705. doi: 10.1371/journal.pone.0188705 (PMC5708723; doi:10.1371/journal.pone.0188705)

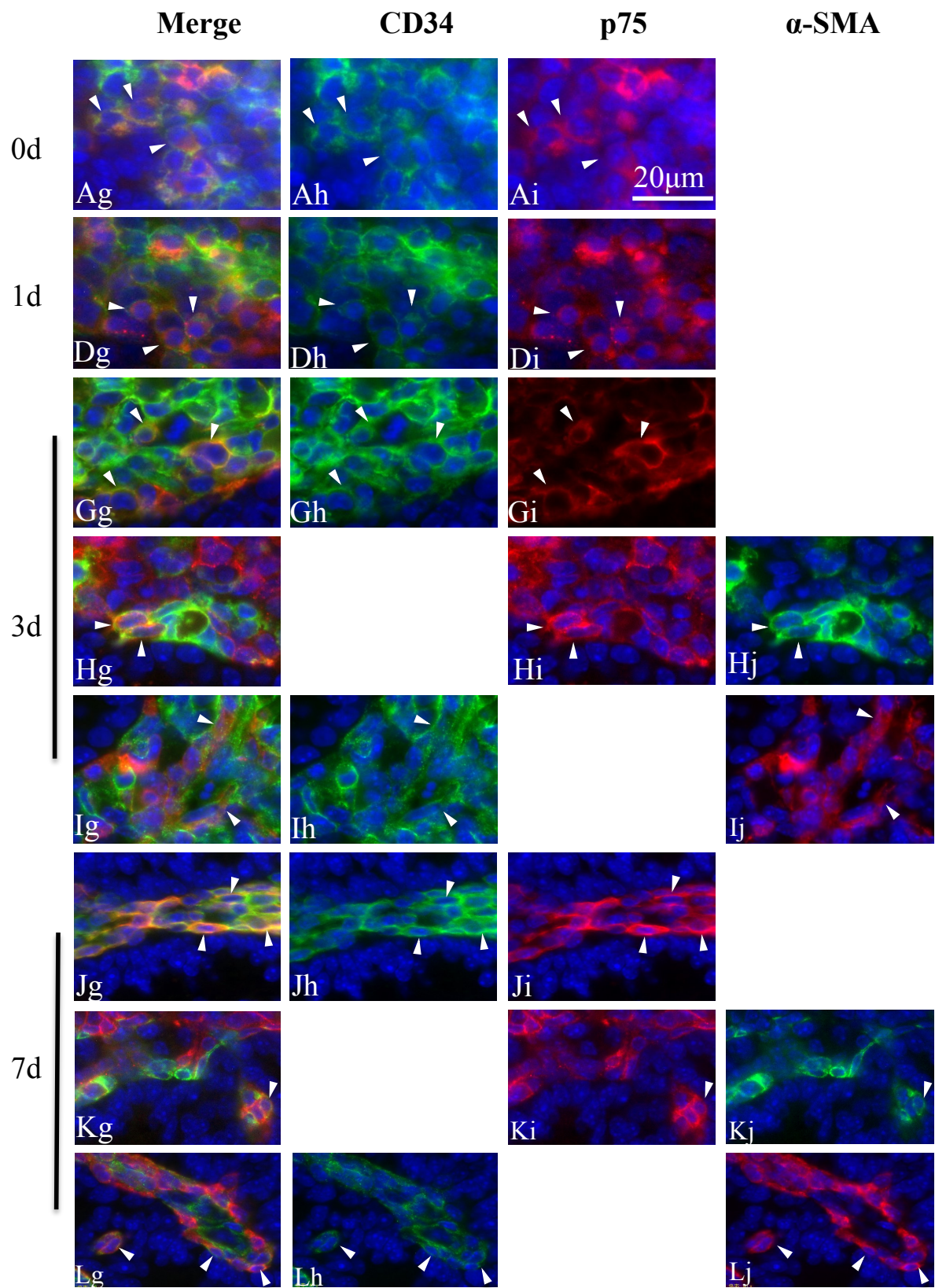

Supplement: S1 Fig — Merged figures (Ag and Dg) with DAPI in the same area as those in Aa and Da (areas encircled by white dotted lines), respectively, in Fig 1 and their resolved figures into CD34 (Ah and Dh) and p75 (Ai and Di) with DAPI were shown. Also merged figures (Gg, Hg, Ig, Jg, Kg, and Lg) with DAPI in the same area as those encircled by white dotted lines in Ga, Ha, Ia, Ja, Ka, and La, respectively, in Fig 2 and their resolved figures into CD34, p75 and α-SMA with DAPI were shown in each row. White arrowheads show cells that expressed two antigens simultaneously. (PDF) [file pone.0188705.s001.pdf]

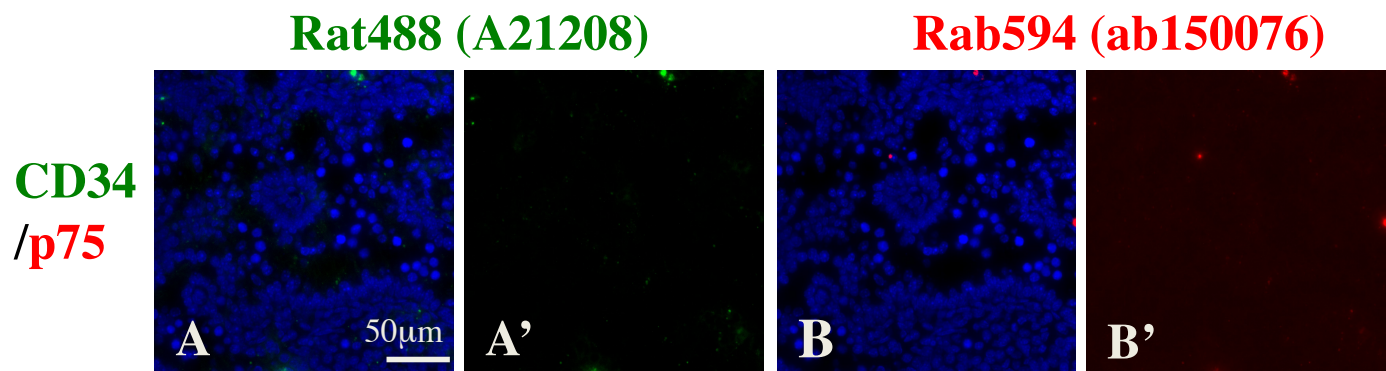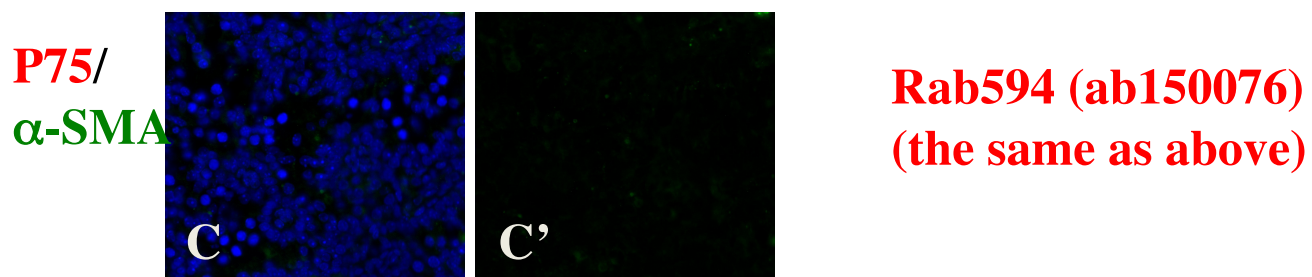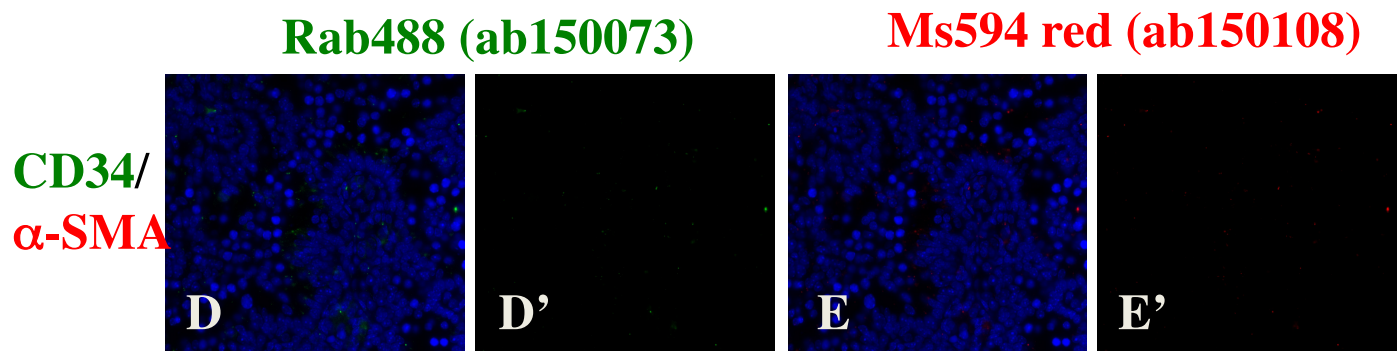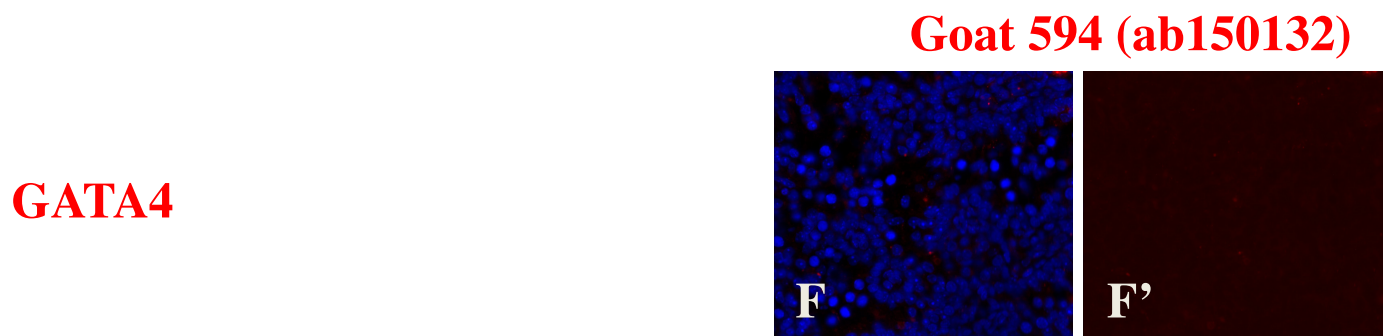

Supplement: S2 Fig — A) A’) D) D’) Negative controls without first antibody against CD34 were shown in A (with DAPI), A’ (without DAPI), D (with DAPI) and D’ (without DAPI). A and A’ were stained only by the secondary antibody, A21208, and D and D’ were stained only by the secondary antibody, ab150073. B) B’) Negative controls for p75 antibody stained by only secondary antibody, ab150076. C) C’) E) E’) Negative controls for α-SMA antibody stained by only secondary antibodies, ab150105 and ab150108. F) F’) Negative controls for GATA4 antibody stained by only secondary antibody, ab150132. (PDF) [file pone.0188705.s002.pdf]

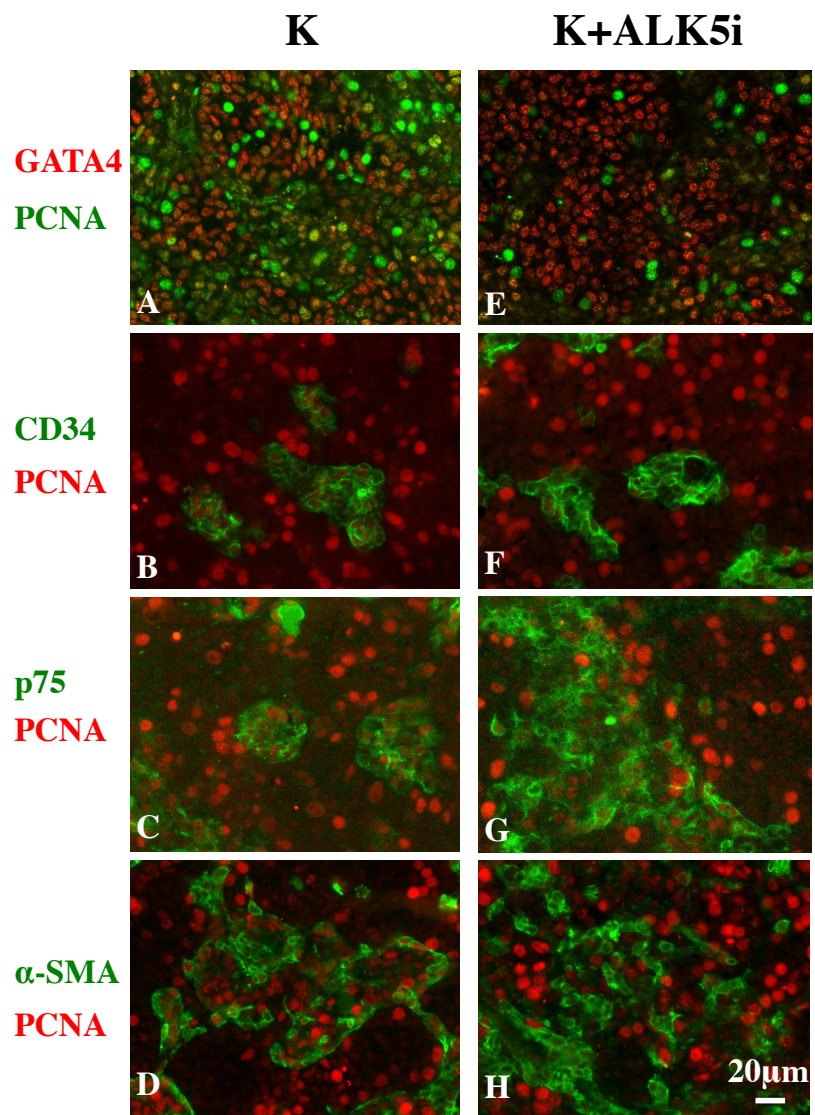

Supplement: S3 Fig — (A)~(D) Double immunostaining with antibodies against PCNA (green) and GATA-4 (red), and antibodies against PCNA (red) and CD34 (green), p75 (green), or α-SMA (green) in the presence of KSR on day 3 of culture. (E)~(H) Double immunostaining with antibodies against PCNA (green) and GATA-4 (red) (E), and antibodies against PCNA (red) and CD34 (green) (F), p75 (green) (G), or α-SMA (green) (H) in the presence of KSR + ALK5i on day 3 of culture. (PDF) [file pone.0188705.s003.pdf]

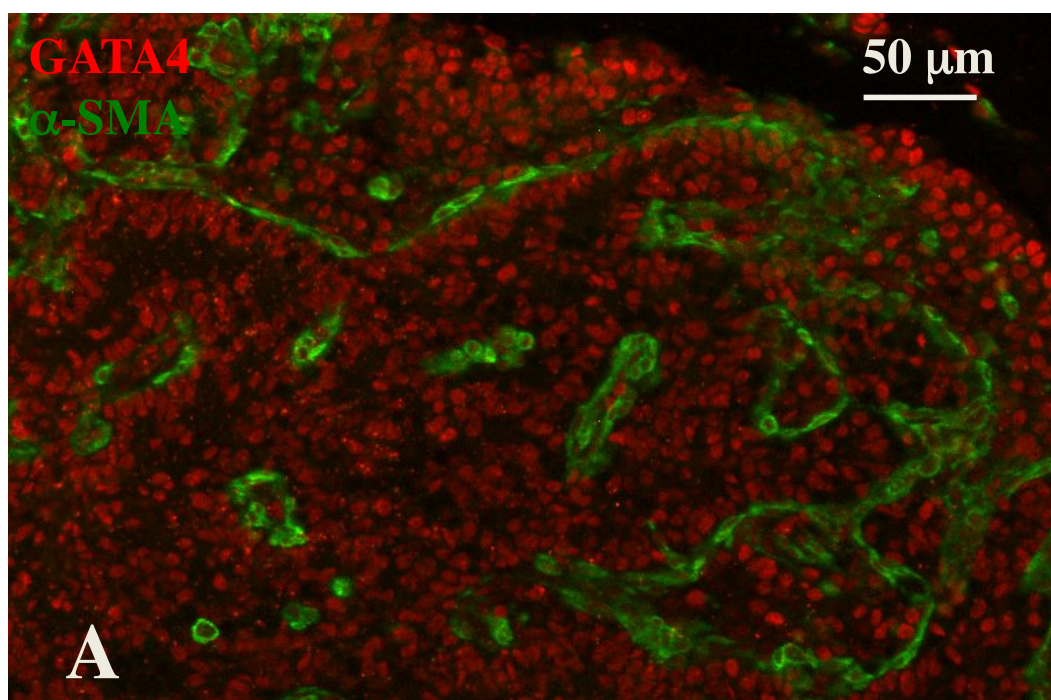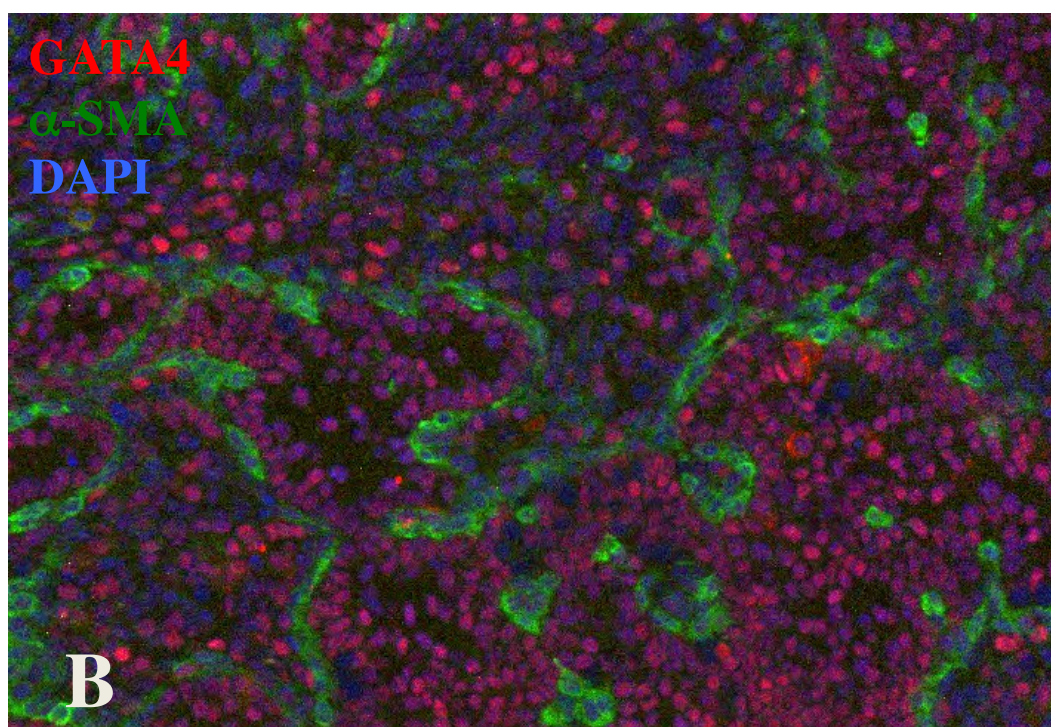

Supplement: S4 Fig — A) Cultured for 5 days. B) Cultured for 7 days. (PDF) [file pone.0188705.s004.pdf]

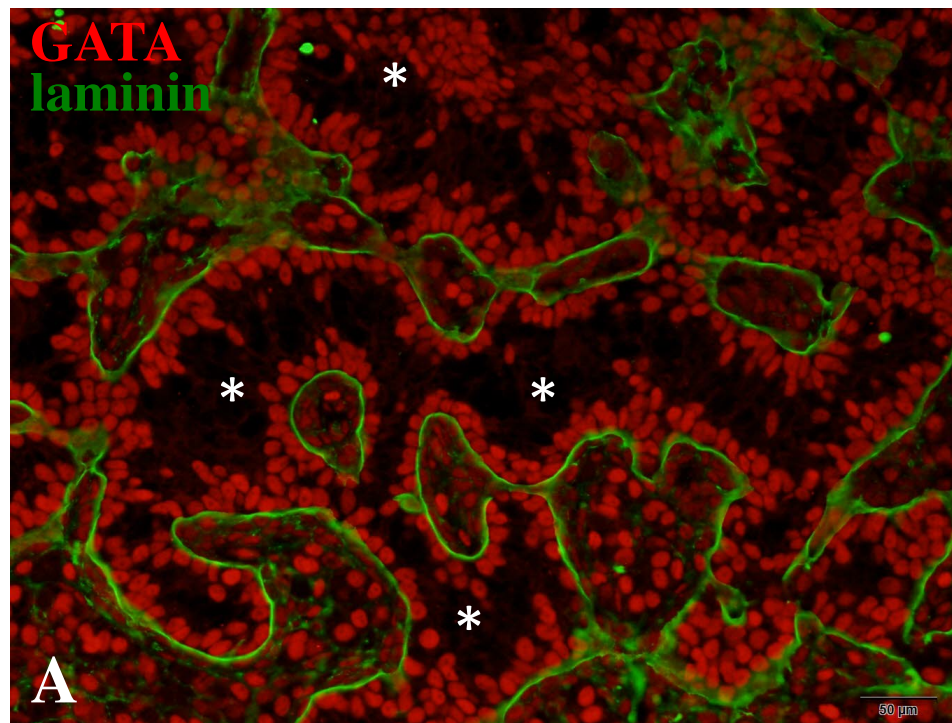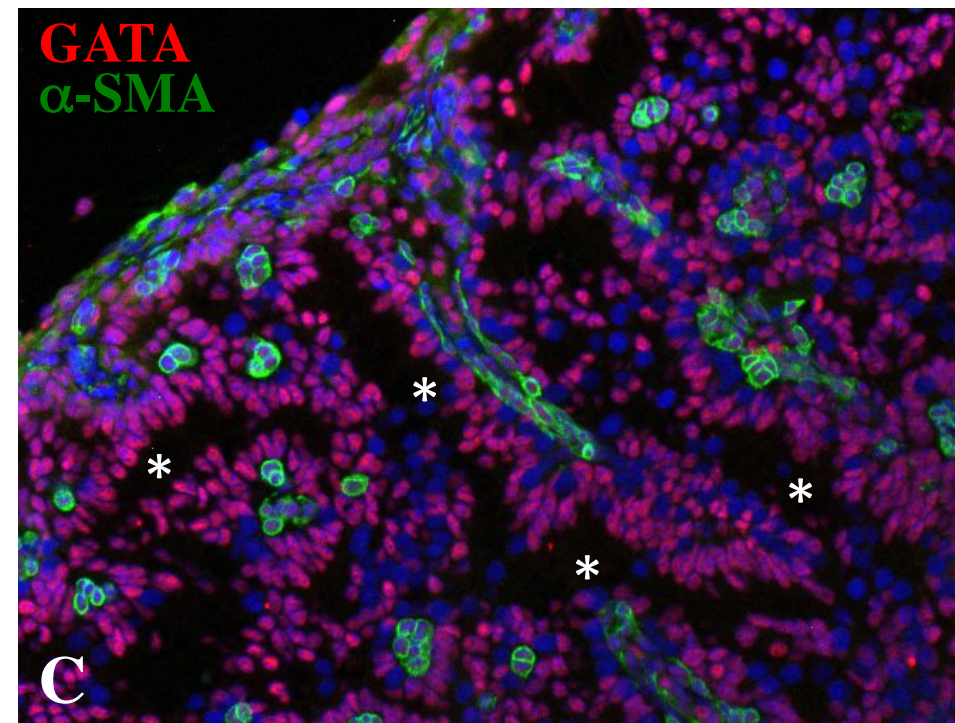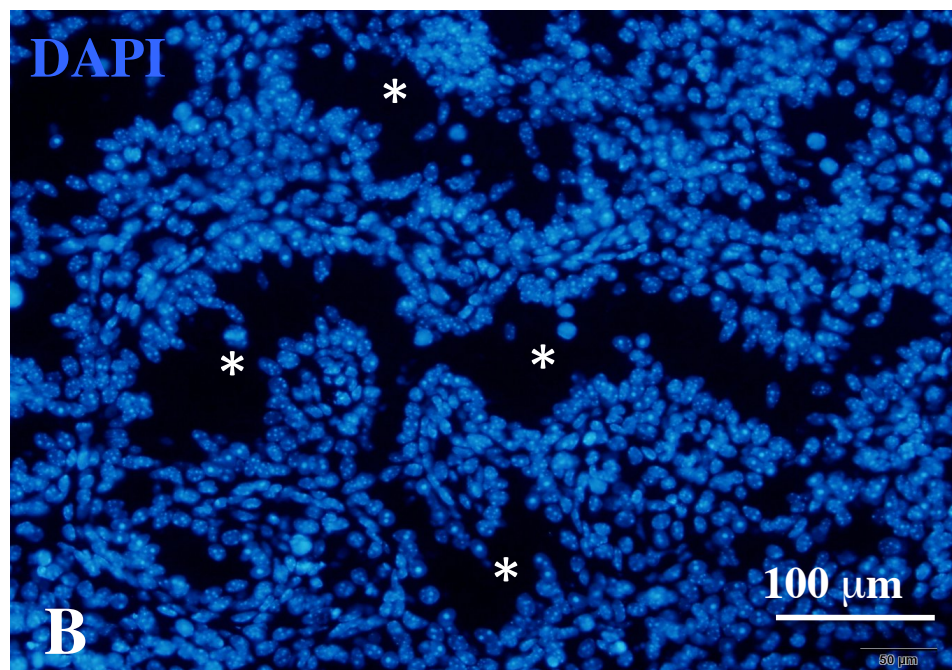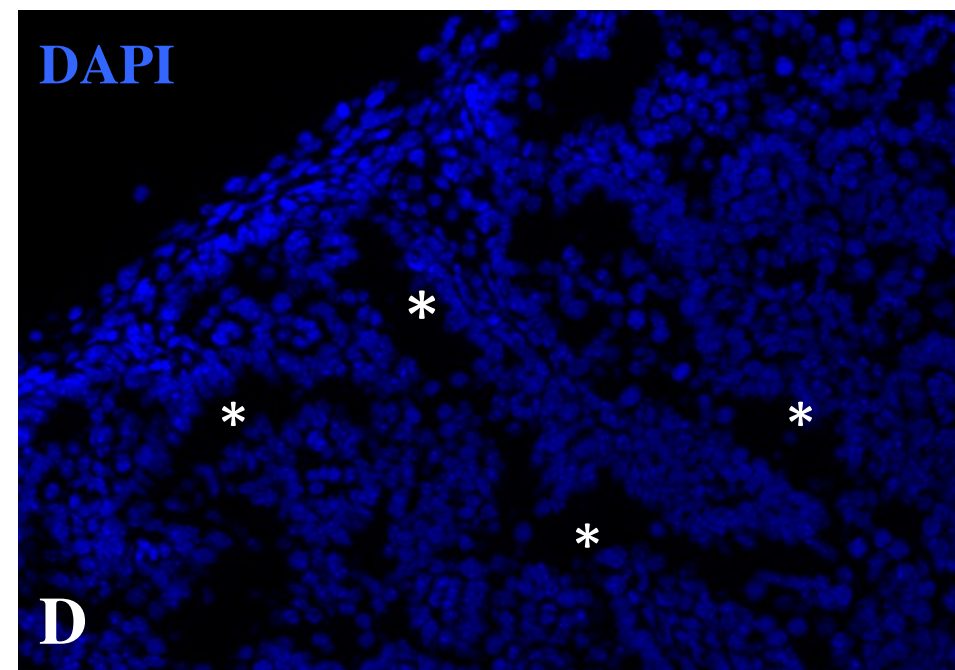

Supplement: S5 Fig — (A) and (C) Double immunostaining of sections cultured for 7 days in the presence of KSR with antibodies against GATA-4 (red) and laminin (green) (A) or α-SMA (green) (C). (B) and (D) Same sections as (A) and (C), respectively, stained with DAPI. (*) shows lumen structures. (PDF) [file pone.0188705.s005.pdf]

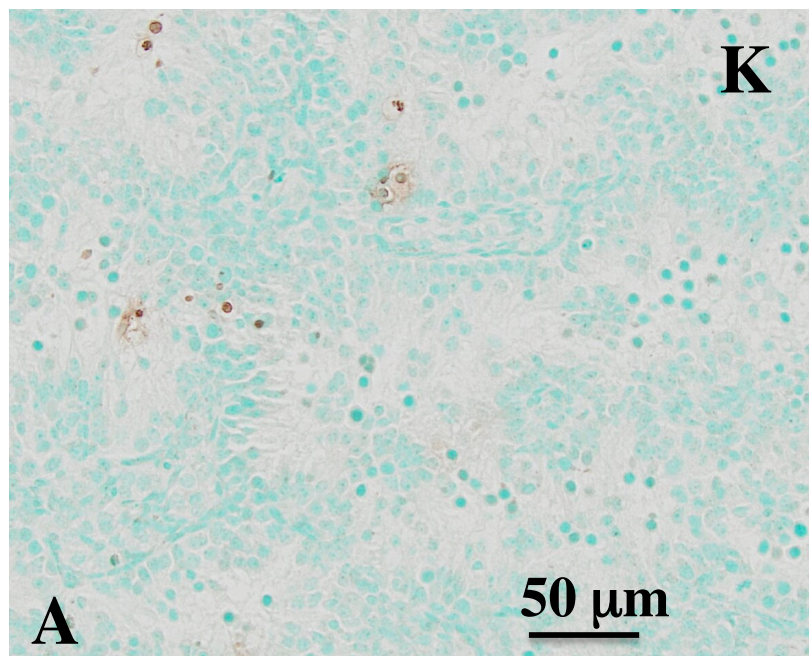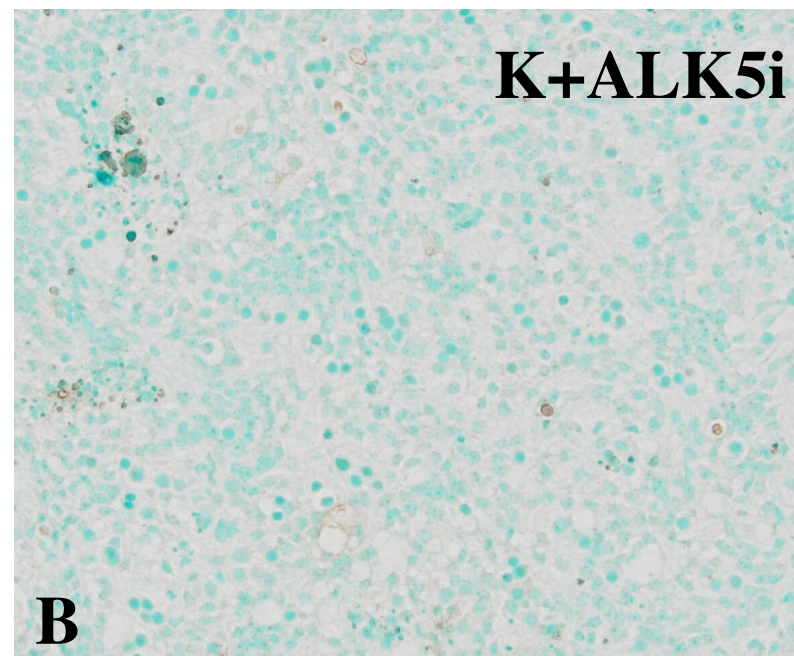

Supplement: S6 Fig — Sections from re-aggregates cultured for 7 days in the absence (A) and presence (B) of 15 ∝M ALK5i were stained with TUNEL and methyl green. (PDF) [file pone.0188705.s006.pdf]

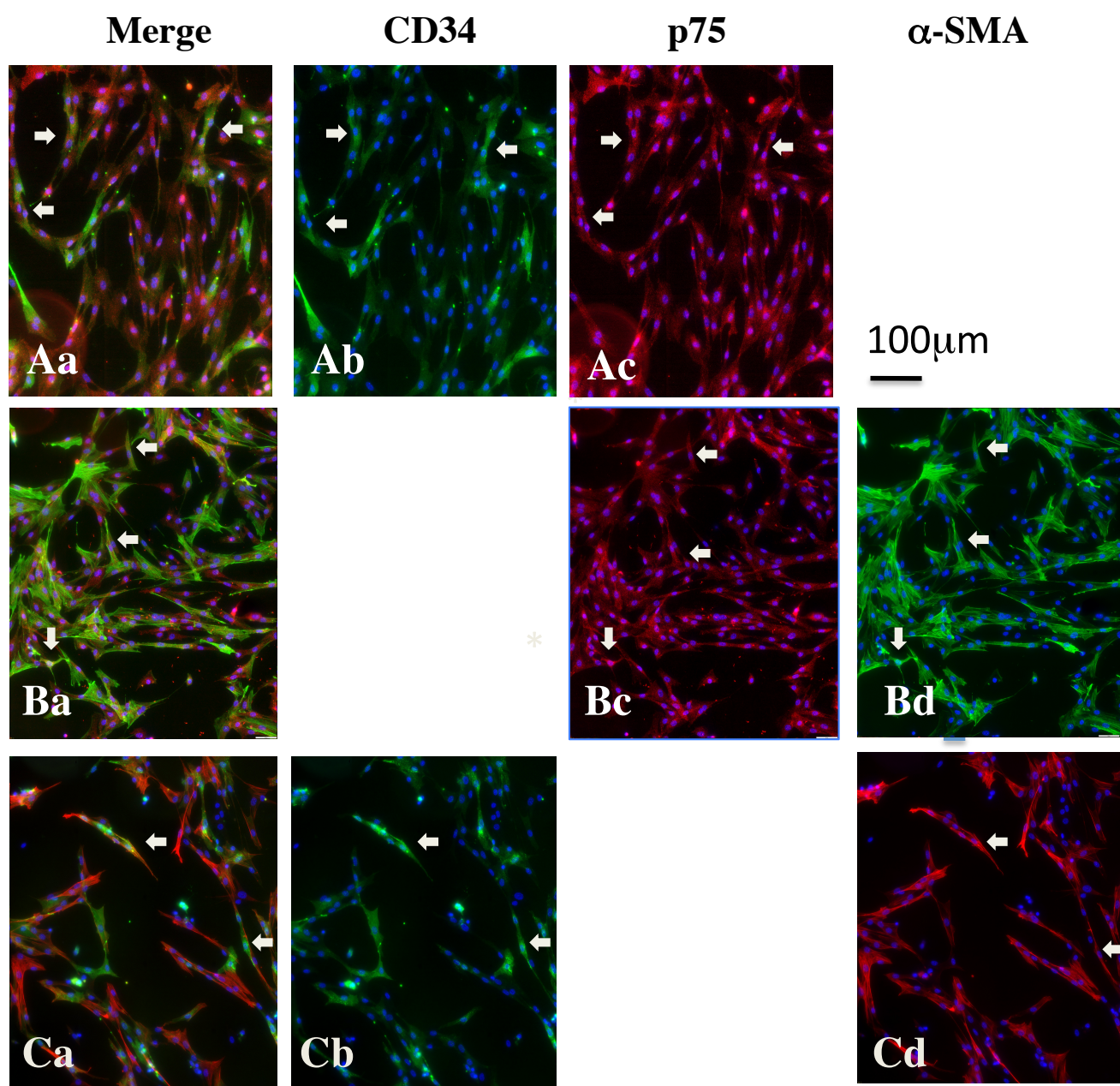

Supplement: S7 Fig — Cells showing both CD34 and p75 (Aa ~ Ac), those double-positive for p75 and α-SMA (Ba~Bd), and those expressing both CD34 and α-SMA are indicated by white arrows. (PDF) [file pone.0188705.s007.pdf]
